# The Microbiological Burden of Short-Term Catheter Reuse in Individuals with Spinal Cord Injury: A Prospective Study

**DOI:** 10.3390/biomedicines11071929

**Published:** 2023-07-07

**Authors:** Tiev Miller, Dirk Lange, Jayachandran N. Kizhakkedathu, Kai Yu, Demian Felix, Soshi Samejima, Claire Shackleton, Raza N. Malik, Rahul Sachdeva, Matthias Walter, Andrei V. Krassioukov

**Affiliations:** 1 International Collaboration on Repair Discoveries (ICORD), Faculty of Medicine, University of British Columbia (UBC), Vancouver, BC V6T 1Z3, Canada; tmiller@icord.org (T.M.); soshi@icord.org (S.S.); shackleton@icord.org (C.S.); razamalik@icord.org (R.N.M.); sachdeva@icord.org (R.S.); 2 Division of Physical Medicine and Rehabilitation, Faculty of Medicine, University of British Columbia (UBC), Vancouver, BC V6T 1Z3, Canada; 3 Department of Urologic Sciences, Faculty of Medicine, University of British Columbia (UBC), Vancouver, BC V6T 1Z3, Canada; dirk.lange@ubc.ca (D.L.);; 4 The Stone Centre at Vancouver General Hospital, Vancouver, BC V5Z 1M9, Canada; 5 Department of Pathology and Laboratory Medicine, Faculty of Medicine, University of British Columbia (UBC), Vancouver, BC V6T 1Z3, Canada; jay@pathology.ubc.ca (J.N.K.); kai.yu@ubc.ca (K.Y.); 6 Centre for Blood Research, Life Science Institute, Faculty of Medicine, University of British Columbia (UBC), Vancouver, BC V6T 1Z3, Canada; 7 The School of Biomedical Engineering, Faculty of Medicine, University of British Columbia (UBC), Vancouver, BC V6T 1Z3, Canada; 8 Department of Urology, University Hospital Basel, University of Basel, 4031 Basel, Switzerland; 9 GF Strong Rehabilitation Centre, Vancouver Coastal Health, Vancouver, BC V5Z 2G9, Canada

**Keywords:** intermittent urethral catheterization, urinary tract infections, bacteria, asymptomatic colonization, electron microscopy, photoelectron spectroscopy, lower urinary tract symptoms, neurogenic urinary bladder disorder, spinal cord injury

## Abstract

Despite the risk of developing catheter-associated urinary tract infections (CAUTI), catheter reuse is common among people with spinal cord injury (SCI). This study examined the microbiological burden and catheter surface changes associated with short-term reuse. Ten individuals with chronic SCI reused their catheters over 3 days. Urine and catheter swab cultures were collected daily for analysis. Scanning electron microscopy (SEM) and X-ray photoelectron spectroscopy (XPS) analyses were used to assess catheter surface changes. Catheter swab cultures showed no growth after 48 h (47.8%), skin flora (28.9%), mixed flora (17.8%), or bacterial growth (5.5%). Asymptomatic bacteriuria was found for most participants at baseline (n = 9) and all at follow-up (n = 10). Urine samples contained *Escherichia coli* (58%), *Klebsiella pneumoniae* (30%), *Enterococcus faecalis* (26%), *Acinetobacter calcoaceticus–baumannii* (10%), *Pseudomonas aeruginosa* (6%) or *Proteus vulgaris* (2%). Most urine cultures showed resistance to one or more antibiotics (62%). SEM images demonstrated structural damage, biofilm and/or bacteria on all reused catheter surfaces. XPS analyses also confirmed the deposition of bacterial biofilm on reused catheters. Catheter surface changes and the presence of antibiotic-resistant bacteria were evident following short-term reuse, which may increase susceptibility to CAUTI in individuals with SCI despite asymptomatic bacteriuria.

## 1. Introduction

Neurogenic lower urinary tract dysfunction (NLUTD) is common in individuals with spinal cord injury (SCI) [1,2]. Detrusor overactivity, detrusor–sphincter dyssynergia, hydronephrosis, reoccurring urinary tract infections (UTI), and the development of bladder and kidney stones are identified sequelae of NLUTD [3]. In people with SCI, several of these associated complications are also established triggers of episodic bouts of hypertension known as autonomic dysreflexia (AD) [4,5]. When left unmanaged or misdiagnosed, AD can result in life-threatening cardiovascular events (e.g., stroke, myocardial infarction) [6,7,8]. Hence, effective lower urinary tract (LUT) management is an important healthcare priority in this population with significant quality of life implications [9,10].

For individuals with SCI, intermittent catheterization is used to manage NLUTD, reduce the incidence of associated complications (e.g., UTI), and improve overall quality of life [11,12]. Single-use intermittent catheterization is considered the current gold standard for individuals with sufficient manual dexterity who are unable to void and require assisted bladder emptying after SCI [1,13,14,15]. Moreover, clinical guidelines currently support single-use catheterization over reuse [16]. Structural damage [17] associated with reuse may cause urethral and bladder tissue trauma and subsequent bacterial growth [18,19], thereby predisposing users to catheter-associated (CA)UTI [1,13,17,20,21]. Despite these known risks, catheter reuse among individuals with SCI is common [21,22,23,24,25,26]. Associated costs [23], resource limitations [26], environmental concerns [22] and convenience [9] are among the principle reasons for reuse. However, evidence supporting the safety of catheter reuse remains inconclusive [27]. A recent case-series involving individuals with SCI investigating the microbial burden associated with reusing hydrophilic polyvinyl chloride catheters found bacterial colonization, encrustation, debris accumulation and structural degradation on reused catheters [28]. These findings suggest subsequent handling and storage of catheters following initial contamination may promote a vicious cycle of further bacterial colonization and persistent biofilm formation [21] which reintroduces bacteria to the LUT upon repeated catheter insertion and removal. This is worrisome, as the associated urothelial damage caused by structural degradation of the catheter surface [17], combined with the development of mature biofilms that preserve adherent bacterial populations against bactericidal agents [29] may increase the risk of developing complicated UTI (e.g., antibiotic resistant infections, extensive systemic infection beyond the LUT), which are potentially difficult to treat [30]. Previous studies investigating catheter reuse in people with SCI involved small (n = 3) [28] or homogenous (i.e., exclusively male) [26] participant samples, which may limit generalizability. Additionally, a comprehensive assessment of catheter surface changes (e.g., chemical composition) following reuse is currently lacking. 

This prospective trial aimed to (1) assess the microbiological burden of short-term catheter reuse in people with SCI and (2) to examine catheter surface changes following reuse. Based on the results of the aforementioned pilot case-series [28], we hypothesized that (1) microbial burden would increase with reuse and (2) structural damage, bacterial colonization and biofilm accumulation would be observable on catheter surfaces following 3 consecutive days of reuse. 

## 2. Materials and Methods

### 2.1. Participants

Individuals with SCI using intermittent catheterization to empty their bladder were either recruited through convenience sampling or were contacted via phone and email using an existing database of previous research participants recruited at the International Collaboration on Repair Discoveries (ICORD) or GF Strong Rehabilitation Centre in Vancouver, British Columbia, Canada. All potential participants were screened for inclusion in-person at ICORD based on predetermined criteria. Inclusion criteria: (1) female or male, (2) ≥18 years old, (3) SCI (American Spinal Injury Association Impairment Scale (AIS) classification grades A to D), (4) sufficient hand function for performing intermittent catheterization independently or with assistance from a caregiver and (5) English proficiency required to provide informed consent, and complete all study-related procedures. Exclusion criteria: (1) having any medical condition(s) that would adversely affect participation, (2) inability to self-catheterize, or unable to obtain assistance in order to perform intermittent catheterization, (3) being or having an immediate family member who is also a member of the investigational team, (4) being pregnant, (5) having previously undergone urinary diversion procedures (e.g., bladder augmentation, cystectomy, neobladder, pouch reservoir, ileal conduit, Mitrofanoff appendicovesicostomy, etc.), (6) having an active UTI and (7) recent use of prophylactic antibiotics. After obtaining written informed consent, relevant medical history and demographic information were collected using questionnaires. All experimental procedures are described in detail in the following sections.

### 2.2. Procedures

#### 2.2.1. Assessment of Incontinence-Related Quality of Life

To determine the effect of urinary incontinence on quality of life, the Incontinence-Quality of Life (I-QoL) questionnaire was used (Supplemental Appendix SA). The I-QoL is a 22-item self-reported measure divided into 3 subscales: (1) avoidance and limiting behavior (8 items), (2) psychosocial impact (9 items) and (3) social embarrassment (5 items). Item responses are based on a 5-point scale (i.e., 1 = extremely; 2 = quite a bit; 3 = moderately; 4 = a little; 5 = not at all), with lower scores indicating poorer quality of life due to incontinence [31]. I-QoL subscale and total scores were then transformed to scaled scores (i.e., (sum of all items − lowest possible score)/possible score range × 100) [32]. The I-QoL has demonstrated good internal consistency (Cronbach alpha (α) = 0.79–0.93) in patients with urinary incontinence due to neurogenic detrusor over-activity (SCI: n = 53, multiple sclerosis: n = 6) [33].

#### 2.2.2. Intermittent Catheterization History

A semi-structured questionnaire was used to collect information on the use of intermittent catheterization following SCI. Questions pertained to catheter type, usage frequency, history of UTI, prophylactic antibiotic usage, inflammatory conditions and other relevant complications. A list of all questionnaire items is provided in Supplemental Appendix SB. 

#### 2.2.3. Urine, Swab Culture and Catheter Specimen Collection

**Figure 1:**
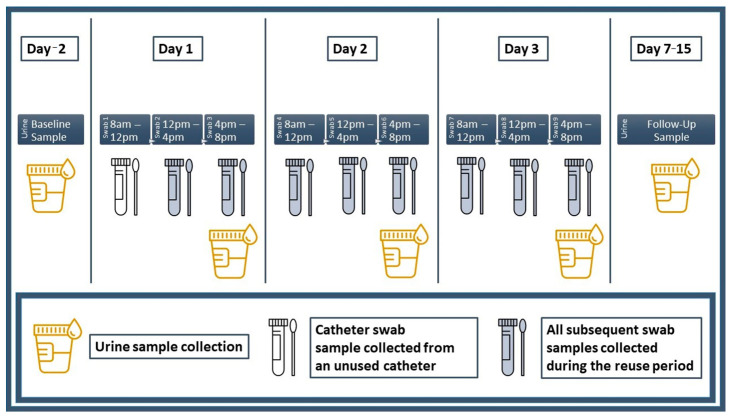
Urine and culture swab collection schedule. Urine and culture swab specimens were collected accordingly as indicated.

Prior to the start of the trial period, participants visited the laboratory and were briefed on urine, culture swab and catheter specimen collection procedures. Participants were then provided with a package containing all study-related materials. A materials summary is provided in Supplemental Appendix SC and a diagram summarizing the collection schedule for all urine and culture swab specimens is provided in Figure 1.

#### 2.2.4. Swab Gram Smear and Culture Analyses

Gram smear and primary culture analyses (n = 10) were performed by a medical laboratory company (LifeLabs Medical Laboratory Services, OMERS Corporation, Toronto, ON, Canada) for all catheter swab specimens. A secondary culture analysis using sonication was conducted for a cohort of 3 randomly selected participants who underwent a second round of reuse with a new catheter in order to verify the reproducibility of culture results obtained from catheter swabs during the primary analysis. Participants reused their catheters for a period of 3 (n = 3) and 30 (n = 1) consecutive days. A detailed summary of gram smear and culture analyses is provided in Supplemental Appendix SC. 

#### 2.2.5. Urinalysis

Urine culture and sensitivity analyses were performed at the same laboratory testing facility (LifeLabs Medical Laboratory Services, OMERS Corp., Toronto, ON, Canada). A detailed summary of urinalysis procedures is provided in Supplemental Appendix SC.

#### 2.2.6. Scanning Electron Microscopy Imaging

Scanning electron microscopy (SEM) imaging of all catheter samples was performed using either a Hitachi SU-3500 system (Hitachi High Technologies Corporation, Tokyo, Japan) or Helios NanoLab 650 Focused Ion Beam system (FEI, Thermo Fisher Scientific, Waltham, MA, USA) at a 1 kV accelerating voltage. For the primary analysis involving 10 catheter samples, SEM imaging was conducted at the Centre for High-Throughput Phenogenomics at the University of British Columbia, Vancouver, BC, Canada. As variance in electron microscopy imaging techniques and interpretation between laboratory settings is expected [34], a secondary analysis was conducted for catheter samples from the same cohort of participants to examine the reproducibility SEM results following a period of 3 (n = 3) and 30 (n = 1) consecutive days of catheter reuse. SEM imaging for these samples was performed using a separate Hitachi SU-3500 system (Hitachi High Technologies Corporation, Tokyo, Japan) located at the University of British Columbia. A detailed summary of SEM procedures is provided in Supplemental Appendix SC. 

#### 2.2.7. X-ray Photoelectron Spectroscopy Analysis

Room-temperature X-ray photoelectron spectroscopy (XPS) was used to examine the surface composition of catheters following 3 (n = 3) and 30 (n = 1) consecutive days of reuse. All experiments were performed at the nanoFAB Centre of the University of Alberta using an XPS imaging spectrometer (Kratos Axis Ultra, Kratos Analytical Ltd., Manchester, UK). A detailed summary of XPS procedures is provided in Supplemental Appendix SC.

### 2.3. Statistical Analyses 

All statistical analyses were performed using SPSS software (version 28, IBM Corp. Armonk, NY, USA). Descriptive statistics are reported as frequencies (n), proportions (%), mean ± sd, or ranges (minimum–maximum). Data normality was assessed with Shapiro–Wilk’s tests (*p* ≤ 0.05). Differences in the number of positive catheter swab and urine specimens at each collection stage (i.e., time point) were assessed using Kruskal–Wallis (H) and Pearson’s χ^2^ tests for continuous and nominal data, respectively (*p* ≤ 0.05) [35,36]. Catheter surface changes determined using SEM and XPS analyses are described narratively.

## 3. Results

### 3.1. Participant Characteristics and Intermittent Catheterization History

A total of 10 participants [females = 5, males = 5, age = 61.6 ± 9.6 years, neurological level of injury: C6–L3, AIS A–D] completed the study. A summary of participant demographics, injury characteristics, incontinence and daily catheter use is provided in Table 1. No symptoms or prophylactic antibiotic use were reported during the course of the study (i.e., from baseline to follow-up). I-QoL scores were relatively lower for Domain 3 (i.e., social embarrassment caused by incontinence) than other scale domains. Participants reported cost (n = 9), convenience (n = 5) or reducing waste/environmental concerns (n = 4) as reasons for reusing catheters. Several participants also reported receiving reuse advise (e.g., cleaning methods) from healthcare providers (n = 4). Most participants reused non-hydrophilic catheters (n = 8). Techniques described for cleaning/preparing catheters for reuse included the use of cold chemical methods (e.g., cold water, soap, hydrogen peroxide, 70% isopropyl alcohol, bleach, vinegar) (n = 7), heat treatment (e.g., hot water) (n = 3), mechanical methods (e.g., washing machine, wiping with a sterile pad) (n = 2) and various storage methods (e.g., containers filled with solution, plastic bag, paper towel wrapping, original packaging) (n = 6). Half of participants reported having a UTI one or more times within the past year (n = 5) and used prophylactic measures to avoid getting UTIs (e.g., antibiotics, herbal supplements) (n = 5). Several participants also reported increased fluid intake as a potential strategy for resolving UTIs (n = 3). No genitourinary conditions (i.e., inflammation of the epididymis, testicles, prostate or pelvis) were reported. A list of individual participant characteristics is provided in Supplemental Table S1. Intermittent catheterization history and questionnaire responses for each participant are summarized in Supplemental Table S2. 

### 3.2. Catheter Swab Gram Smear, Culture and Sensitivity

A summary of gram smear, culture and sensitivity analysis results for all catheter swab specimens (i.e., total of 90) is provided in Supplemental Table S3. Time point comparisons are summarized in Table 2. Catheter swab gram stains taken during the 3-day reuse period showed the presence of epithelial cells (n = 9, 25/90, 27.8%), gram-positive bacilli (n = 8, 13/90, 14.4%), gram-negative bacilli (n = 3, 8/90, 8.9%), gram-positive cocci (n = 4, 7/90, 7.8%) and neutrophils (n = 6, 10/90, 11.1%). Swab gram stain cellularity did not differ between time points (χ^2^ = 12.000, *p* = 0.062) (Table 2). 

Catheter swab cultures showed either no growth after 48 h (n = 10, 43/90, 47.8%), contained skin flora (n = 8, 26/90, 28.9%) or mixed flora (n = 4, 16/90, 17.8%) or showed *Escherichia coli* (n = 2, 5/90, 5.6%), *Klebsiella pneumoniae* (n = 1, 1/90, 1.1%), *Acinetobacter* species (n = 1, 1/90, 1.1%), or *Staphylococcus aureus* (n = 1, 1/90, 1.1%) and *Staphylococcus lugdunensis* growth (n = 1, 1/90, 1.1%). Less than half of bacterial cultures demonstrated antibiotic resistance (n = 2, 2/90, 2.2%). There were no differences in the proportion of positive culture results (χ^2^ = 10.819, *p* = 0.094) or antibiotic resistant cultures (χ^2^ = 4.286, *p* = 0.117) between time points (Table 2). For the secondary culture analysis, results showed a bacterial growth of 40 million (M) colony forming units (CFU)/L for one catheter sample following 3 days of reuse (Participant 3). No bacterial growth was observed for other catheter samples analyzed (Supplemental Table S4).

### 3.3. Urinalysis 

A summary of urine chemistry analysis results for all urine specimens (i.e., total of 50) is provided in Supplemental Table S5. Urine color was yellow for most specimens (n = 10, 46/50, 92%) and dark yellow for a few samples (n = 2, 4/50, 8%). Urine was either clear (n = 10, 33/50, 66%), cloudy (n = 7, 12/50, 24%) or turbid (n = 3, 5/50, 10%) in appearance. Urine pH was within normal range (5.0–8.0) for all specimens except one (≥9.0). Urine specific gravity was within normal range (1.003–1.035) for most specimens (n = 9, 39/50, 78%), with the remainder showing low specific gravity (≤1.005) (n = 4, 11/50, 22%), possibly indicative of urine dilution due to high fluid intake prior to sample collection. All urine protein results (<0.3 g/L) were negative except one (0.3 g/L). Urine glucose results were negative for all specimens (<2.8 mmol/L). All urine ketone results were negative (<0.5 mmol/L) except one (1.5 mmol/L). Urine hemoglobin was negative (<0.3 mg/L) for most specimens (n = 9, 38/50, 76%), with the remainder showing positive results (ranging from trace amounts to 80 mg/L) (n = 5, 12/50, 24%) suggesting hematuria. Urine nitrite was negative for most specimens (n = 10, 29/50, 58%), with just under half showing positive urine nitrite results (n = 9, 21/50, 42%). Urine leukocytes were present in most specimens (ranging from 70 to 500 white blood cells (WBC)/μL) (n = 10, 40/50, 80%), with negligible amounts (<25 WBC/μL) observed in the remainder (n = 3, 10/50, 20%). No significant time point differences were observed for all urine chemistry results (Table 2).

Urine culture and sensitivity analysis results are summarized in Supplemental Table S6. Asymptomatic bacteriuria (ASB) was found for most participants at baseline (n = 9) and all participants at follow-up (n = 10). Urine specimens contained *Escherichia coli* (n = 6, 29/50, 58%), *Klebsiella pneumoniae* (n = 3, 15/50, 30%), *Enterococcus faecalis* (n = 3, 13/50, 26%), *Acinetobacter calcoaceticus–baumannii* complex or *pittii* cultures (n = 1, 5/50, 10%), *Pseudomonas aeruginosa* (n = 1, 3/50, 6%) or *Proteus vulgaris* (n = 1, 1/50, 2%) with >100 M CFU/L for most cultures (n = 10, 43/50, 86%). Most urine specimen bacterial cultures showed resistance to one or more antibiotics (n = 8, 31/50, 62%). There were no differences in the proportion of positive urine cultures (χ^2^ = 3.125, *p* = 0.537) or antibiotic-resistant urine cultures (χ^2^ = 0.340, *p* = 0.987) between time points (Table 2).

### 3.4. Scanning Electron Microscopy Images

**Figure 2:**
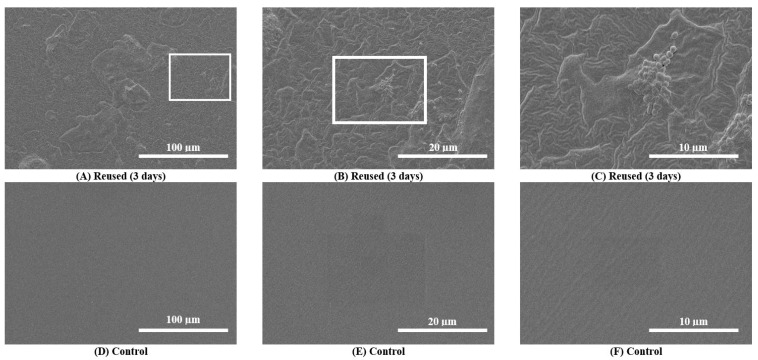
Scanning electron microscopy (SEM) images of the outer surfaces of a reused catheter and pristine control catheter. (**A**–**C**): SEM images of the outer surfaces of catheter samples after 3 consecutive days of reuse (upper panel images) for a single participant (Participant 3). Surface damage, debris accumulation and bacterial colonization on the reused catheter are observable at progressive magnifications (500× to 5000×). (**D**–**F**): SEM images of the outer surfaces of the pristine control catheter sample do not show any damage.

Scanning electron microscopy (SEM) images taken during the primary analysis for 10 catheter samples demonstrated catheter surface damage, debris accumulation and bacterial colonization (i.e., visible bacilli and cocci) in and around catheter eyelets as well as the inner lumen and outer catheter surfaces after 3 consecutive days of reuse (n = 10) (Supplemental Figures S1–S3). SEM images taken during the secondary analysis (n = 3) also showed surface damage, debris accumulation and bacterial colonization (Figure 2). These features were absent on pristine control catheters (Figure 2). For Participant 6, debris and biofilm accumulation found on the outer surface of the 30-day sample (Supplemental Figure S4A–C) was relatively greater than the 3-day sample (Supplemental Figure S4D–F).

### 3.5. X-ray Photoelectron Spectroscopy Analysis

**Figure 3:**
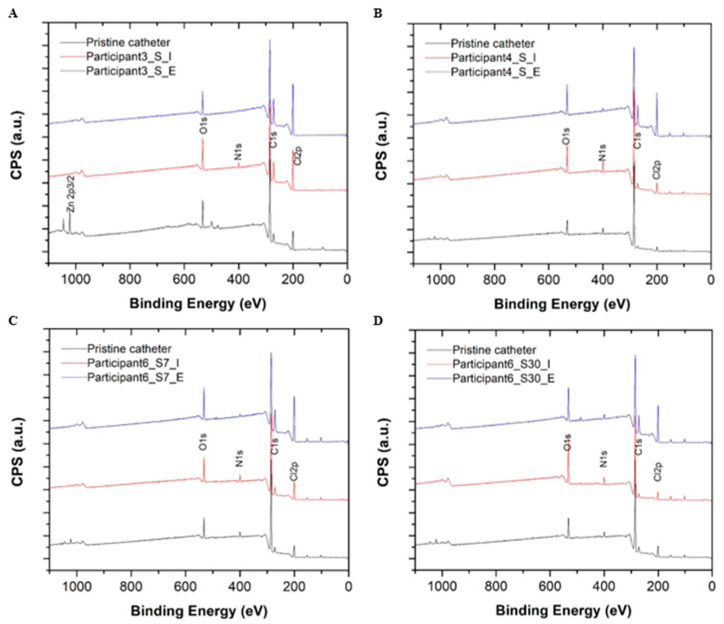
High resolution X-ray photoelectron spectroscopy (XPS) survey scans for the pristine control catheters (black) and the intralaminar (red) and extraluminal (blue) catheter surfaces after 3 days of reuse by Participant 3 (**A**), Participant 4 (**B**), and Participant 6 (**C**) are depicted above. XPS survey scans for a pristine control catheter (black) and the intralaminar (red) and extraluminal (blue) catheter surfaces after 30 days of reuse by Participant 6 are also depicted (**D**). Abbreviations: C = carbon, CPS = counts per unit, eV = electron volts, N = nitrogen, O = oxygen.

A summary of the catheter surface composition analysis performed using high-resolution X-ray photoelectron spectroscopy (XPS) is provided in Table 3. A quantitative summary of carbon peak decomposition for different chemical bonding environments during the XPS survey scan is provided in Figure 3. Compared to pristine control catheter surfaces, all reused samples showed an enrichment of nitrogen (N) and oxygen (O) on catheter surfaces suggesting mostly organic material deposition (i.e., bacterial colonization and biofilm) for two chemical bonding environments (i.e., =C-NH, NH2-C-COOH, where nitrogen is more likely to be bonded to carbon (C) or oxygen). No metal ions or phosphor deposits were detected on the surfaces of reused catheters during the XPS survey scan indicating an absence of salt deposition. The carbon (C)1s high resolution scan presented additional evidence of the debris and biofilm composition on the surfaces of reused catheters in comparison to pristine controls. The carbon decomposed into four peaks, ranging from 284.6 eV to 289 eV. The area of each decomposed peak matched the carbon percentage during different chemical bonding environments. Each peak was situated at 285.6 eV and 286.5 eV, which corresponded to carbon adjacent to nitrogen (Figure 3). The increase observed in these components also suggests organic debris and biofilm deposition on reused catheter surfaces but not pristine controls (Supplemental Figure S5). The amount of deposition on intralaminar surfaces differed from extraluminal surfaces (Sample S4 vs. S5; S6 vs. S7; S8 vs. S9; S10 vs. S11) (Table 3, Supplemental Figure S5). For Participant 6, composition percentage for carbon bonded to nitrogen (C1s at 285.6 eV and 286.5 eV) was relatively higher for the 30-day sample than the 3-day sample on both the intralaminar (S10 vs. S8) and extraluminal surfaces (S11 vs. S9) indicating reuse duration influenced the amount of organic debris and biofilm deposition (Table 3, Supplemental Figure S5).

## 4. Discussion

### 4.1. Evidence Summary

This observational trial adds to the limited body of literature on the microbiological burden associated with catheter reuse in individuals with SCI and NLUTD [26,28]. Our findings are largely consistent with a previous pilot trial suggesting microbiological burden may increase with daily reuse [28]. ASB was also observed for 9/10 participants at baseline, and all participants during the trial period and at follow-up, which exceeds current prevalence estimates for this population (i.e., individuals with SCI using CIC = 23–69%, or with sphincterotomy and condom catheters = 57%) [30]. Despite clinical guidelines supporting single-use [16] and the potential risk of developing a complicated infection, 4/10 participants reported receiving reuse advise from healthcare providers after their injury. During the reuse period, a total of eight types of bacteria were identified from catheter swab and urine sample specimens within a relatively short period of time (i.e., 72 h). While all organisms demonstrated antibiotic susceptibility, most organisms also showed intermediate or full resistance to several common antibiotics (e.g., ampicillin, ciprofloxacin, clindamycin, erythromycin, tetracycline, etc.). Antibiotic resistance may increase the frequency of subsequent CAUTI, which poses a significant barrier to treatment due to the limited out-patient therapies available [30,37]. This is cause for concern, as genitourinary complications such as recurring urosepsis, represent a substantial healthcare burden (e.g., an estimated annual cost of USD 2.8 billion in the US alone) [37], and remain a major cause of morbidity and mortality (i.e., 16.2%) among people living with SCI [38,39]. Moreover, noxious stimuli which trigger episodic AD are caused by genitourinary sources (e.g., bladder distension, UTI) in up to 85% of people with SCI [40,41]. Hence, recurring CAUTI may increase the frequency and severity of AD and exacerbate cardiovascular morbidity and mortality risk [6,7,8].

### 4.2. Chemistry, Cellularity and Bacterial Growth 

Catheter swab gram stains showed the presence of epithelial cells for almost all participants (n = 9, 27.8%), potentially suggesting catheter-associated urethral trauma sustained by these individuals at some point during the trial. Urothelial cell shedding is also indicative of bacterial infection in individuals with chronic LUT symptoms [42,43]. Leukocytes, a marker of systemic inflammation, were found in most urine specimens (n = 10, 80%) while positive nitrite, a metabolic by-product of potentially pathogenic origin, was observed in just under half of urine specimens (n = 9, 42%). The detection of both leukocytes and nitrite for two or more consecutive urine specimens potentially increases the probability of developing CAUTI (likelihood ratio = 1.0–10.6) [44]. This trend was observed among half (n = 5) of the study participants despite ASB. While a large proportion of swab cultures were negative for bacterial growth, many showed skin (28.9%) or mixed flora (17.8%). Although samples containing mixed flora are often dismissed in other populations (e.g., elderly) [45,46], polymicrobial contamination may be indicative of individual differences in catheterization technique, catheter handling, cleaning (e.g., use of bactericidal agents) and/or storage methods between catheterizations in people with SCI [28]. Of the organisms identified during the trial, *Escherichia coli* and *Klebsiella pneumoniae* were the most common and are known to cause the majority of symptomatic UTIs [47]. These were found in both swab cultures and urine sample specimens. Other organisms of potential pathogenic importance include *Proteus vulgaris* found in one urine sample, and *Enterococcus faecalis* found in several urine samples [47]. UTIs caused by *Enterococcus faecalis* have demonstrated persistent growth and rapid antibiotic resistance making infections particularly difficult to eradicate with conventional treatment [43]. Other organisms identified in urine samples (i.e., *Acinetobacter calcoaceticus–baumannii* complex or *pittii* cultures, *Pseudomonas aeruginosa*) and swab culture specimens (i.e., *Acinetobacter* species, *Staphylococcus aureus* and *Staphylococcus lugdunensis*) are known to cause UTIs in hospital and long-term care settings [48,49]. *Pseudomonas aeruginosa* is also known to exhibit relatively greater antibiotic resistance and biofilm formation on medical devices than either *Escherichia coli* or *Klebsiella pneumonia* [50]. 

### 4.3. Catheter Surface Changes 

More extensive surface damage (e.g., uneven, porous or abraded surfaces) is likely to promote bacterial adhesion, colonization and biofilm formation [51]. Once formed, mature biofilms may serve as an extracellular insulation for adherent bacterial colonies against chemical cleaning and bactericidal agents [29]. Previous evidence suggests cold chemical soaking solutions are effective in sterilizing catheters and preserving their structural integrity after one reuse [52,53]. Although several participants in the current study reported using soaking solutions between catheterizations (n = 6) (i.e., liquid soap, liquid bleach, hydrogen peroxide, 70% isopropyl alcohol), the results of the surface imaging and composition analyses suggest relatively longer reuse periods (i.e., 3 or more consecutive days) caused catheter surface damage, debris and biofilm deposition, and bacterial colonization. These findings are consistent with previous catheter reuse trials of varying durations (i.e., 3 days, average of 21 days, average of 35 months) which also demonstrated outer surface and inner lumen encrustation [26,28], biofilm, debris and bacterial contamination on reused catheters [21]. To our knowledge, this is the first study to examine the chemical composition of reused catheter surfaces using XPS analysis. Our results suggest relatively greater biofilm deposition on the outer surface and inner lumen following 30 days of reuse in comparison to 3 days. Future studies examining progressive (i.e., daily) changes to surface composition and concomitant bacterial growth are needed moving forward.

### 4.4. Limitations

This study has several limitations. First, catheter reuse patterns and cleaning routines varied considerably between individuals, which may limit the generalizability of the results. Although this variability is perhaps a realistic portrayal of catheter reuse among individuals with SCI, several participants reported that, outside of this trial, they typically rotate catheters between cleaning, storage and reuse. According to a recent systematic review investigating preparation methods for cleaning and reusing catheters [54] only two studies described cold chemical cleaning methods (i.e., soaking catheters in a 0.6% sodium hypochlorite solution, or a 70% alcohol solution after only one reuse) that were effective in sterilizing catheters without incurring structural damage [52,53]. Whether soaking solutions used by the participants in this study (e.g., hydrogen peroxide) could potentially become incorporated with (or alter) the material properties of catheters in a way that either promotes or prevents greater debris accumulation and bacterial colonization is unknown and warrants further investigation. 

Second, our ability to determine progressive differences in swab and urine cultures during the trial was limited by the presence of bacteriuria at baseline for most participants. Previous evidence suggests ASB may even play a protective role in preventing symptomatic infections in female patients with recurrent uncomplicated UTI [55]. Thus, the influence of ASB for either increasing or reducing the risk of complicated UTI in people with SCI and NLUTD remains inconclusive and requires further investigation. Future studies involving standardized catheter cleaning methods and larger participant cohorts who are required to avoid reusing their catheters prior to starting the trial may help to address this limitation. Comparative studies examining the incidence of symptomatic UTI between cohorts practicing single-use or multiple-use intermittent catheterization are also warranted.

Third, only a small proportion of catheter swab specimens showed bacterial growth (n = 4, 8/90, 8.9%) bringing into question the necessity of collecting more than one sample per day. Future studies involving longer follow-up periods may benefit from collecting single rather than multiple daily swab samples. 

Fourth, in preparing catheter samples for SEM imaging, debris, biofilm and bacteria on catheter surfaces may have also been displaced/disturbed/dislodged during transport, cutting and/or fixation in preparation for analysis, and thus the magnitude of organic deposition on catheter surfaces observed in this study may be under-representative.

Lastly, compared to other neurological populations with NLUTD, the prevalence of SCI is relatively lower (i.e., 41–68 persons per million). Clinical trials and case-series involving small participant samples are common in SCI research [56]. The participant sample recruited for this study was also small and comparative analyses between time points were likely underpowered. The study design may also limit the generalizability of the results as participants were recruited using a non-probability sampling method (i.e., convenience) thereby unintentionally excluding individuals with diverse clinical presentations (e.g., lesion location, injury severity). Future studies should involve larger sample sizes to enhance the generalizability of these results.

### 4.5. Conclusions

This trial attempted to provide a realistic picture of the microbiological burden associated with catheter reuse in a cohort of individuals with SCI who presented with NLUTD. Our preliminary findings are consistent with those reported previously, suggesting catheter surface damage, debris and biofilm deposition, and bacterial colonization occurs with consecutive short-term catheter reuse in people with SCI. A number of bacteria were identified from catheter swab and urine samples within a relatively short period of time (i.e., 72 h), most of which showed resistance to one or more antibiotics, thereby increasing the risk of developing CAUTI that are potentially difficult to treat. The presence of urine leukocytes and nitrite in consecutive samples also suggests that half of the participants were at risk of developing CAUTI during the course of the trial. Despite these findings, no incidences of symptomatic UTI were reported. However, our results suggest that, given the right conditions, the risk of developing complicated CAUTI increased significantly among the study participants during the course of the trial. Longer periods of observation during reuse among larger participant cohorts with SCI and NLUTD would allow for a more accurate determination of CAUTI incidence.

## Figures and Tables

**Table 1:** Participant characteristics.

	(N = 10)
Demographics	
Age (years)	61.6 ± 9.6
Age—range (years)	50–79
Height (cm)	173.5 ± 5.9
Weight (kg)	71.9 ± 17.6
BMI (kg/m^2^)	24.0 ± 6.7
Injury Characteristics	
Time since injury (years)	35.6 ± 9.8
Time since injury—range (years)	17–50
Neurological level of injury—range	C6–L3
Motor—complete, AIS (A/B)	5/0
Motor—incomplete, AIS (C/D)	1/4
Daily Catheterization	
Average	5.2 ± 1.7
Average—range	3–9
Minimum	3.6 ± 1.4
Minimum—range	1–6
Maximum	7.9 ± 1.9
Maximum—range	5–10
Catheter Reuse	
Reuse history (years)	29.4 ± 10.7
Reuse history—range (years)	11–44
Shortest reuse time (single catheter) (days)	10.0 ± 14.9
Shortest reuse time—range (single catheter) (days)	1–42
Longest reuse time (single catheter) (days)	71.1 ± 106.4
Longest reuse time—range (single catheter) (days)	1–336
Incontinence	
I-QoL—scaled Domain 1 (0–100)	77.5 ± 14.5
I-QoL—scaled Domain 2 (0–100)	86.9 ± 11.2
I-QoL—scaled Domain 3 (0–100)	73.0 ± 20.6
I-QoL—scaled total (0–100)	80.3 ± 12.6

Values are reported as mean ± sd, range (min–max), or frequencies (n). Abbreviations: AIS = American Spinal Injury Association Impairment Scale, BMI = body mass index, C = cervical, I-QoL = Incontinence-Quality of Life, L = lumbar.

**Table 2:** Time point comparisons.

Variable	Day −2 (Baseline)	Day 1	Day 2	Day 3	Day 7–15 (Follow-Up)	χ^2^	H	*p*
Swab Gram Smear, Culture and Sensitivity								
Gram stain cellularity † (swab # 1/2/3), n (0–10)	-	3/2/1	2/1/5	7/1/0	-	12.000	-	0.062
Positive culture results (swab # 1/2/3), n (0–10)	-	4/2/0	1/2/5	0/3/4	-	10.819	-	0.094
Antibiotic resistance, n (0–10)	-	0	2	0	-	4.286	-	0.117
Urine Chemistry								
Appearance (clear/cloudy/turbid), n (0–10)	6/3/1	8/1/1	6/3/1	7/2/1	6/3/1	1.818	-	0.986
Urine pH (5.0–8.0)	7.10 ± 1.02	6.55 ± 0.98	6.10 ± 0.77	6.55 ± 0.98	6.10 ± 0.77	-	6.679	0.154
Specific gravity (1.003–1.035)	1.012 ± 0.004	1.012 ± 0.006	1.014 ± 0.007	1.013 ± 0.008	1.015 ± 0.005	-	1.616	0.806
Hemoglobin (mg/L)	2.53 ± 7.89	2.50 ± 7.90	2.53 ± 7.89	5.03 ± 10.52	10.56 ± 25.62	-	2.725	0.605
Leukocyte (WBC/μL)	250.00 ± 220.47	182.00 ± 172.37	196.00 ± 213.90	164.00 ± 183.58	189.00 ± 219.55	-	1.542	0.819
Nitrite (positive), n (0–10)	2	3	4	6	6	5.255	-	0.272
Urine Culture and Sensitivity								
Positive culture results, n (0–10)	9	9	10	10	10	3.125	-	0.537
Antibiotic resistance, n (0–10)	6	6	7	6	6	0.340	-	0.987

† Presence of epithelial cells, gram-positive bacilli, gram-negative bacilli, gram-positive cocci, and/or neutrophils. Values are reported as mean ± sd, or frequencies (n).

**Table 3:** X-ray photoelectron spectroscopy results summary.

Sample No.	Participant—Catheter Sample	† C%	N%	O%	‡ Carbon			
					C-C	C-NH_2_, C-N	-C-O, =C-NH, NH_2_-C-COOH	O=C-OH
					284.6 eV	285.6 eV	286.5 eV	288.5–289 eV
S1	Participant 3—pristine control	82.59	0	8.69	87.1	0	9.12	3.78
S2	Participant 4—pristine control	87.14	3.32	5.99	93.15	0	3.72	3.13
S3	Participant 6—pristine control	82.77	2.47	7.28	90.38	0	6.42	3.19
S4	Participant 3—reused catheter (3 days, intralaminar)	72.02	2.35	9.96	61.78	14.41	20.64	3.17
S5	Participant 3—reused catheter (3 days, extraluminal)	72.83	0.15	7.40	66.26	5.83	25.14	2.77
S6	Participant 4—reused catheter (3 days, intralaminar)	80.45	4.8	8.76	72.85	14.25	8.1	4.8
S7	Participant 4—reused catheter (3 days, extraluminal)	69.92	1.25	9.35	62.76	16.22	19.17	1.85
S8	Participant 6—reused catheter (3 days, intralaminar)	77.71	3.02	8.93	77.59	9.34	11.3	1.76
S9	Participant 6—reused catheter (3 days, extraluminal)	68.67	1.29	9.57	64.94	8.1	25.39	1.58
S10	Participant 6—reused catheter (30 days, intralaminar)	74.22	3.42	15.30	68.7	8.71	15.93	6.66
S11	Participant 6—reused catheter (30 days, extraluminal)	70.15	2.12	10.30	57.41	16.94	23.42	2.23
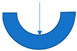	Intralaminar catheter surface	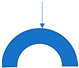		Extraluminal catheter surface				

† Molar percentage of chemical elements (C, N, O) on the surface. ‡ Carbon percentage for each chemical bonding environment (highlighted in red). Abbreviations: C% = percent carbon, N% = percent nitrogen, O% = percent oxygen.

## Data Availability

The data presented in this study are contained within the article and Supplementary Materials. SPSS (version 28, IBM Corp. Armonk, NY, USA) and CASA XPS (Surface Analysis Consultation, Clearwater, FL, USA) are commercially available softwares. No custom code was used.

## Supplementary Materials

The following supporting information can be downloaded at: https://www.mdpi.com/article/10.3390/biomedicines11071929/s1, Supplemental Figure S1. Scanning electron microscopy images (eyelet and inner surface); Supplemental Figure S2. Scanning electron microscopy images (inner surface); Supplemental Figure S3. Scanning electron microscopy images (eyelet and outer surface); Supplemental Figure S4. Scanning electron microscopy images (outer surface of catheters reused for 3 days and 30 days); Supplemental Figure S5. X-ray photoelectron spectroscopy results summary; Supplemental Table S1. Individual participant demographics and injury characteristics; Supplemental Table S2. Semi-structured survey on intermittent catheterization; Supplemental Table S3. Catheter swab gram smear and culture analysis; Supplemental Table S4. Secondary catheter culture analysis; Supplemental Table S5. Urine chemistry analysis; Supplemental Table S6. Urine culture analysis; Supplemental Appendix SA: Incontinence-Quality of Life (I-QoL); Supplemental Appendix SB: Intermittent catheterization questionnaire; Supplemental Appendix SC: Materials, procedures and assessments.
