# Supplementary material for: The Microbiological Burden of Short-Term Catheter Reuse in Individuals with Spinal Cord Injury: A Prospective Study
Source: Biomedicines. 2023 Jul 7;11(7):1929. doi: 10.3390/biomedicines11071929 (PMC10377649; doi:10.3390/biomedicines11071929)
Supplement: Supplementary file 1 [file biomedicines-11-01929-s001.zip › biomedicines-2475165-supplementary.pdf]

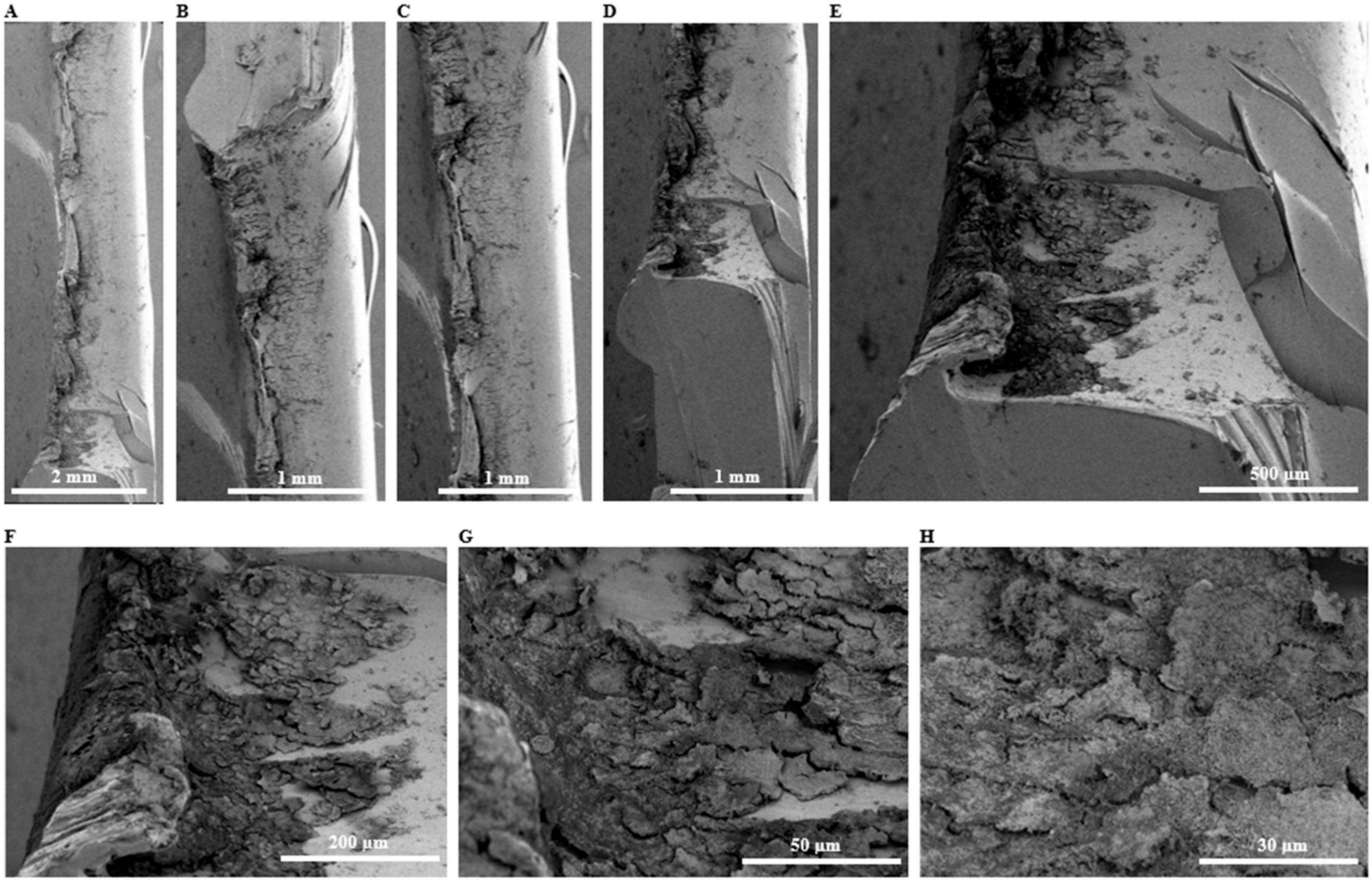

Supplemental Figure S1. Scanning Electron Microscopy (SEM) images of the eyelet and inner lumen surface of a catheter sample after 3 consecutive days of reuse. Catheter surface damage (A-E), and debris accumulation (F-H) were observable at progressive magnifications (25× to 1,500×).

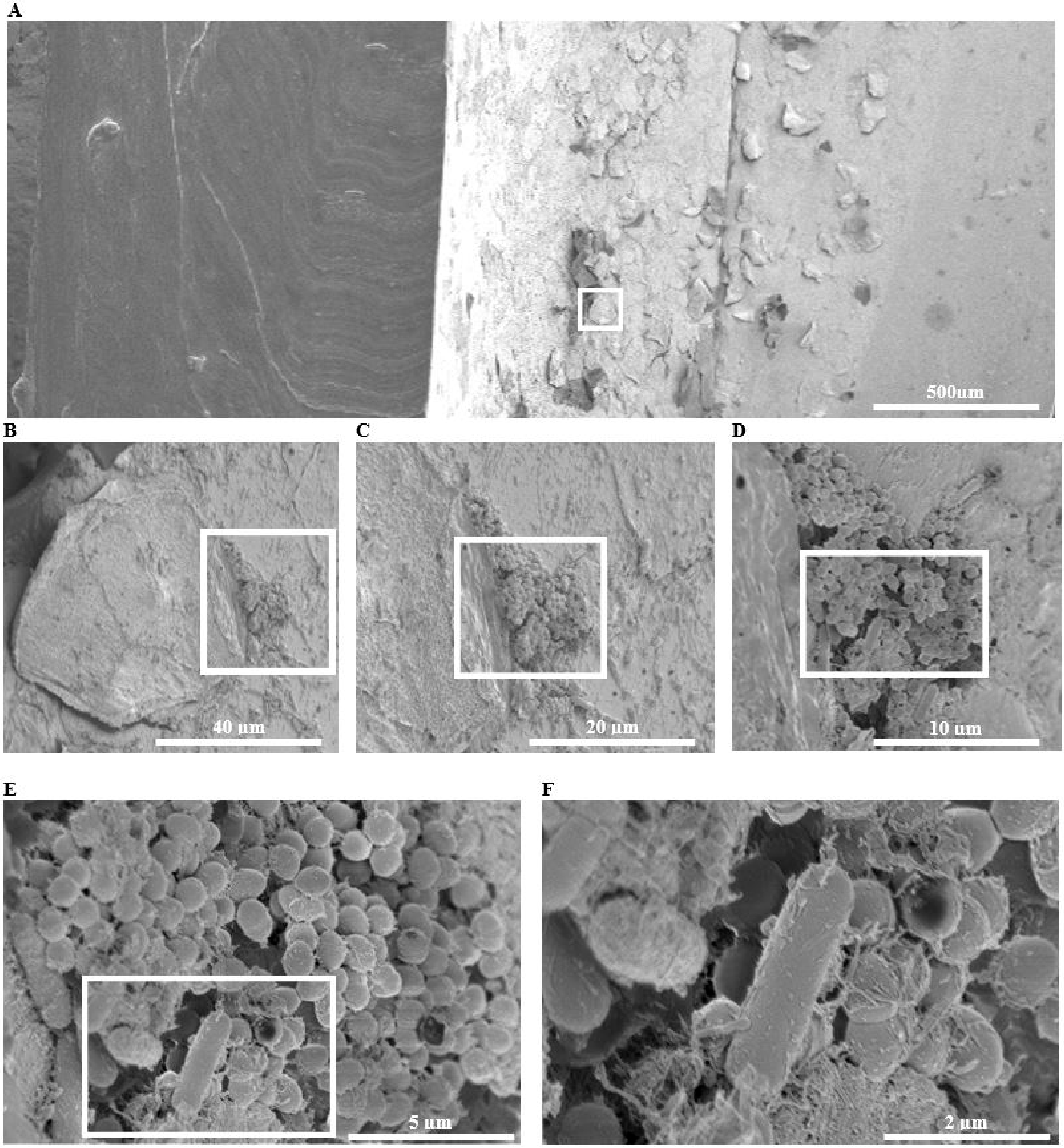

Supplemental Figure S2. Scanning Electron Microscopy (SEM) images of the inner lumen surface of a catheter sample after 3 consecutive days of reuse. Catheter surface damage (A), debris accumulation (B-D) and bacterial colonization (B-F) were observable at progressive magnifications (100× to 24,500×). White boxes in each image represent the area magnified in the subsequent image (A through F).

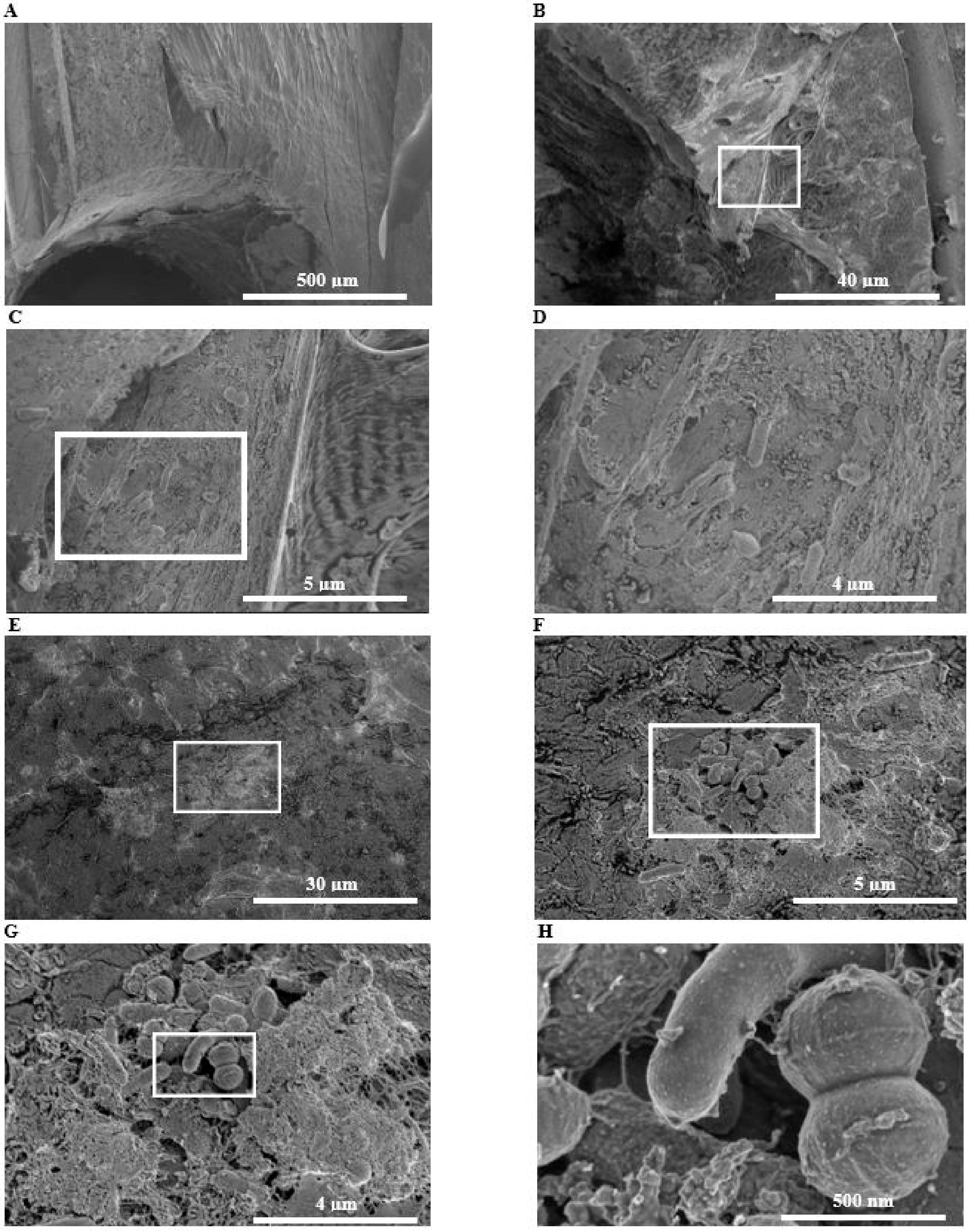

Supplemental Figure S3. Scanning Electron Microscopy (SEM) images of the eyelet and outer surface of a catheter sample after 3 consecutive days of reuse. Catheter surface damage (A-B), debris and biofilm accumulation (C-F) and bacterial colonization (G-H) are observable at progressive magnifications (100× to 63,500×). White boxes in each image represent the area magnified in the subsequent image (A through D, and E through H).

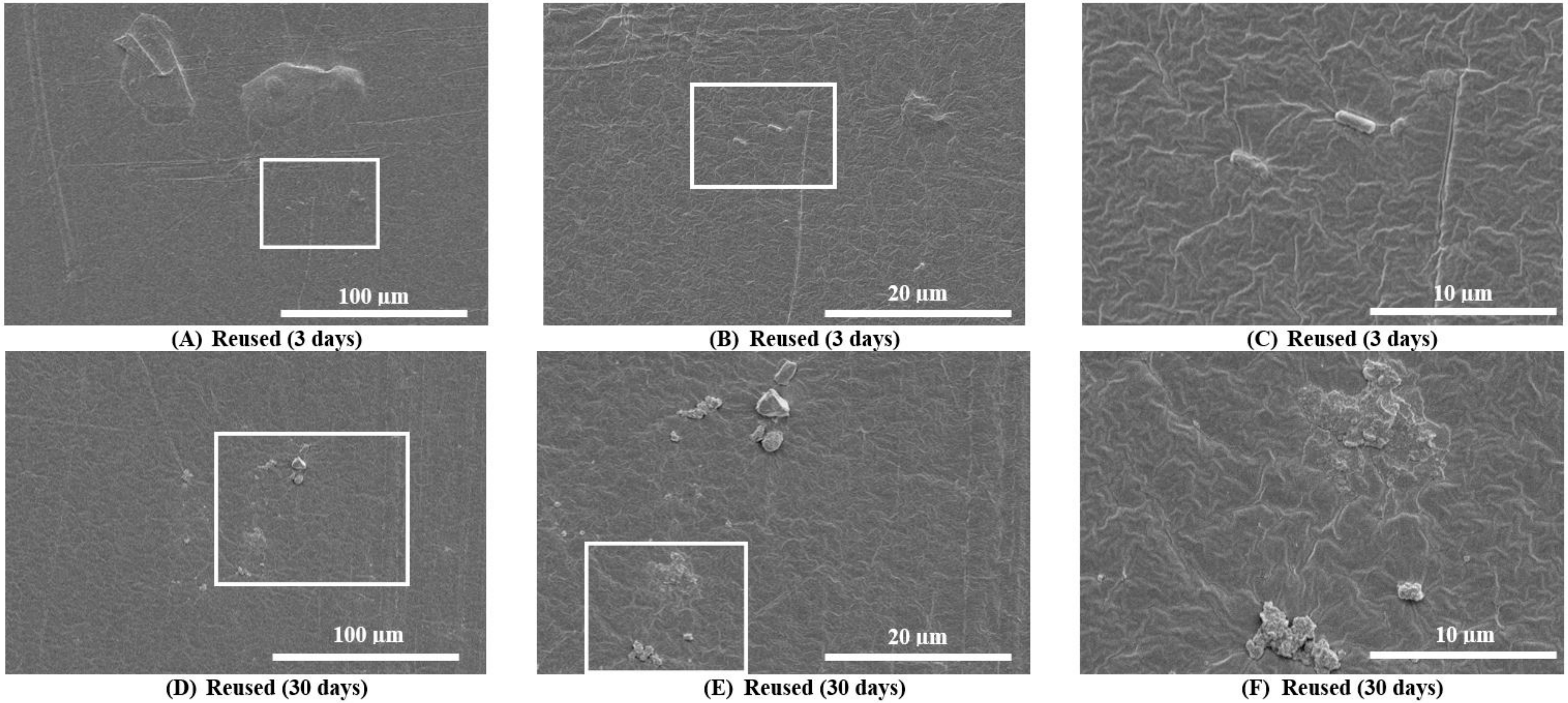

Supplemental Figure S4. Scanning Electron Microscopy (SEM) images of the outer surfaces of catheter samples after 3 (A-C) and 30 consecutive days of reuse (D-F) for a single participant (Participant 6). Catheter surface damage, debris accumulation and bacterial colonization are observable at progressive magnifications (500× to 5,000×). Debris accumulation found on the outer surface of the 30-day sample was relatively greater than the 3-day sample. White boxes in each image represent the area magnified in the subsequent image (A through C, and D through F).

A (S1)

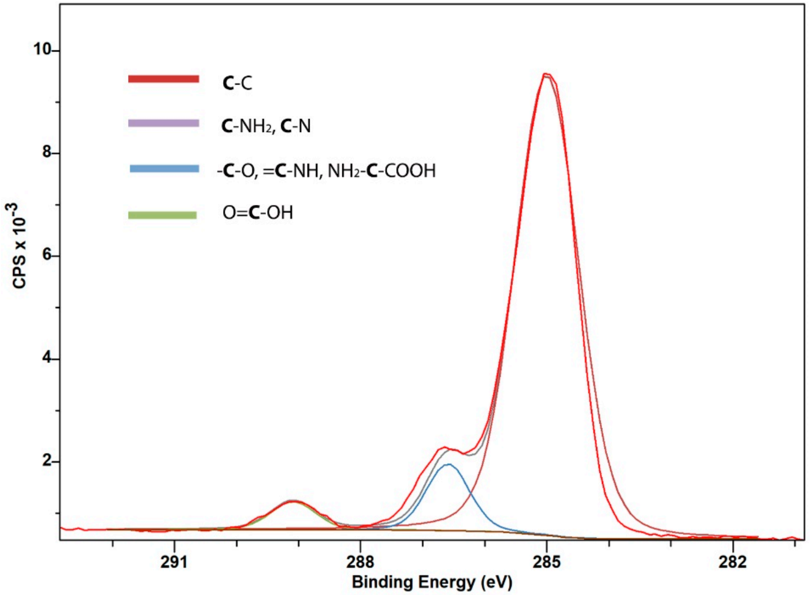

B (S2)

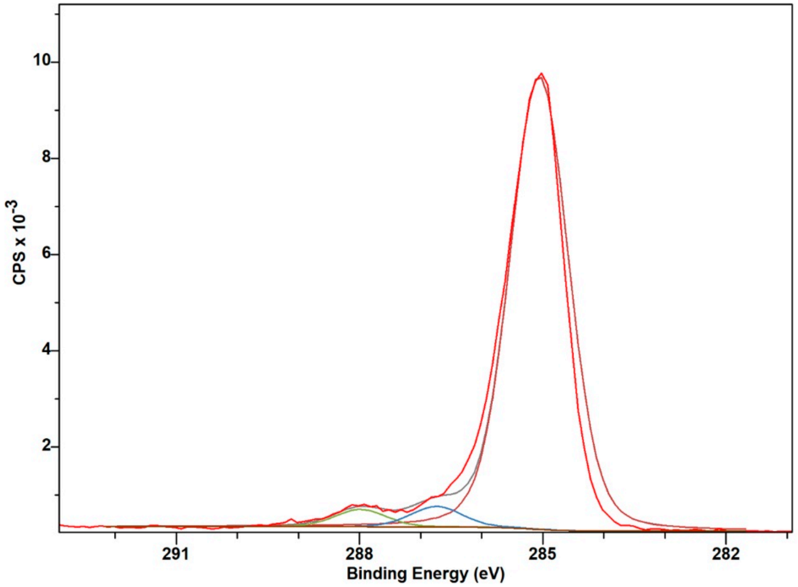

C (S3)

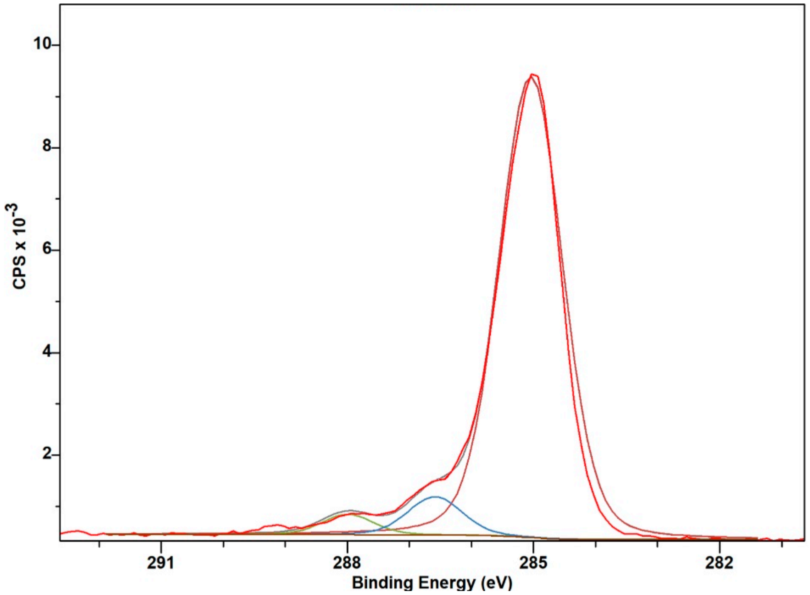

D (S4)

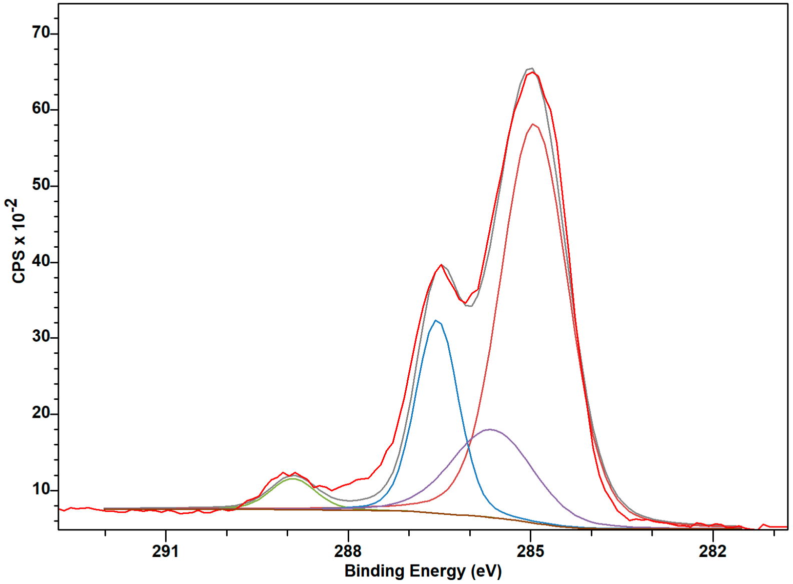

E (S5)

F (S6)

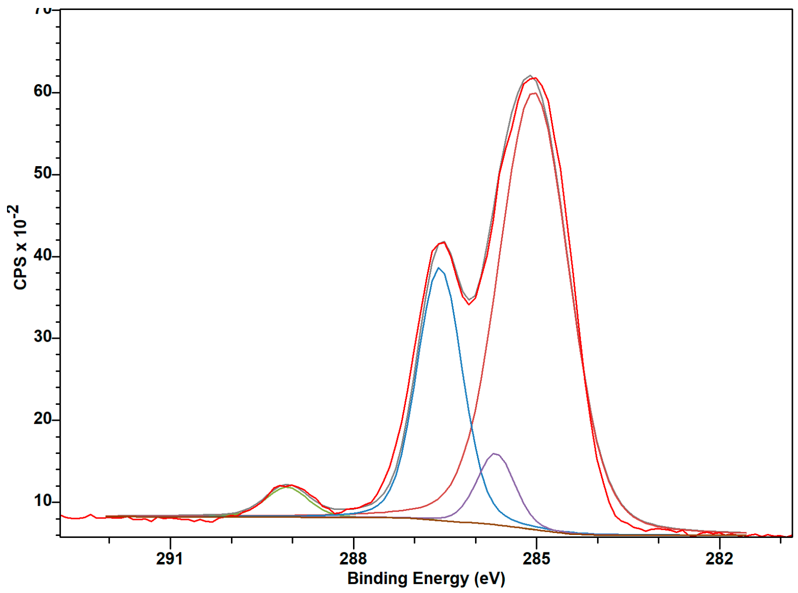

G (S7)

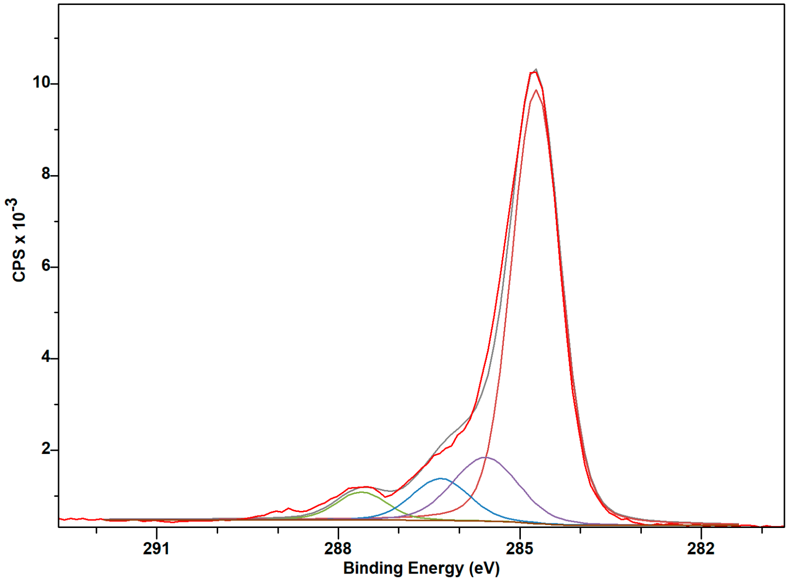

H (S8)

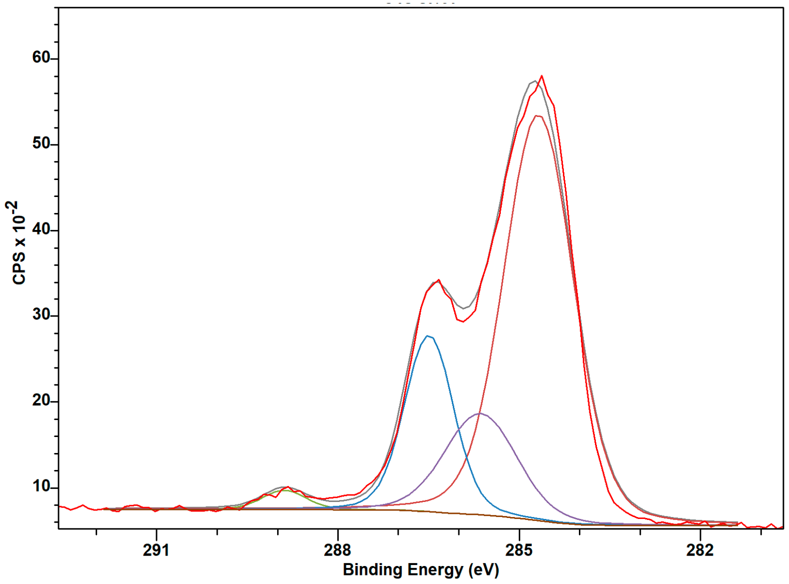

I (S9)

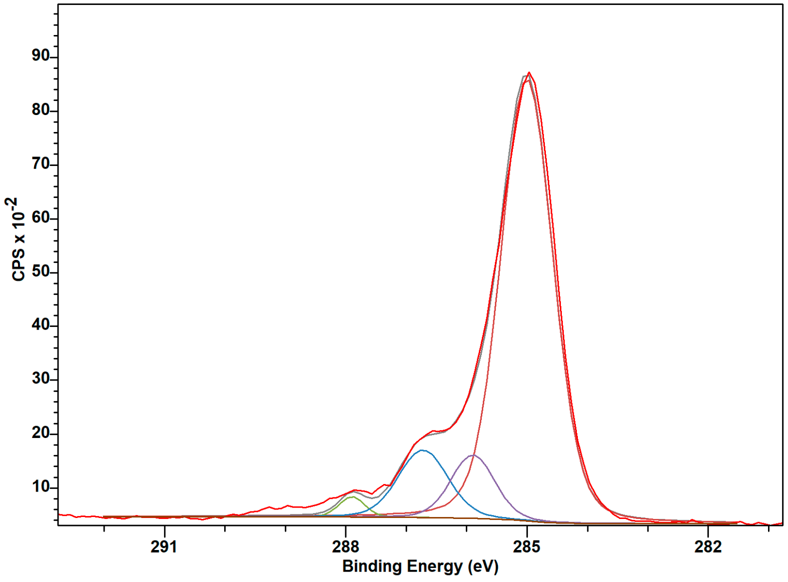

J (S10)

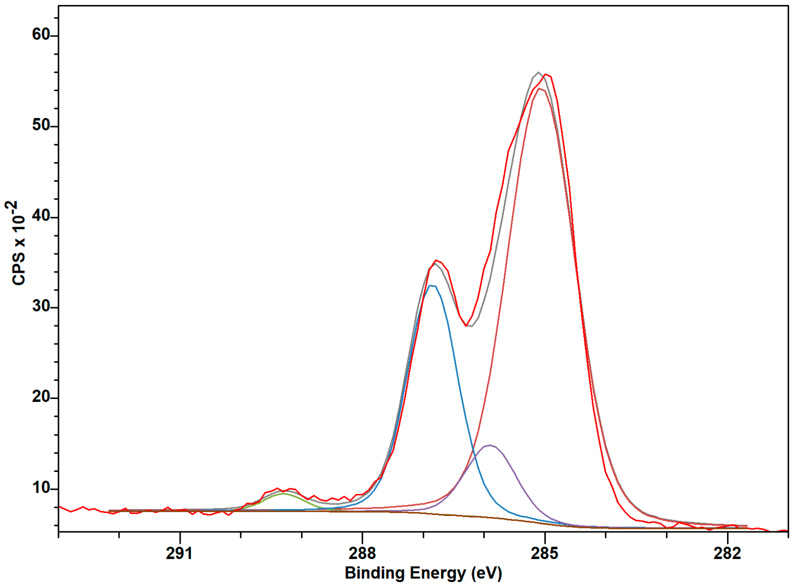

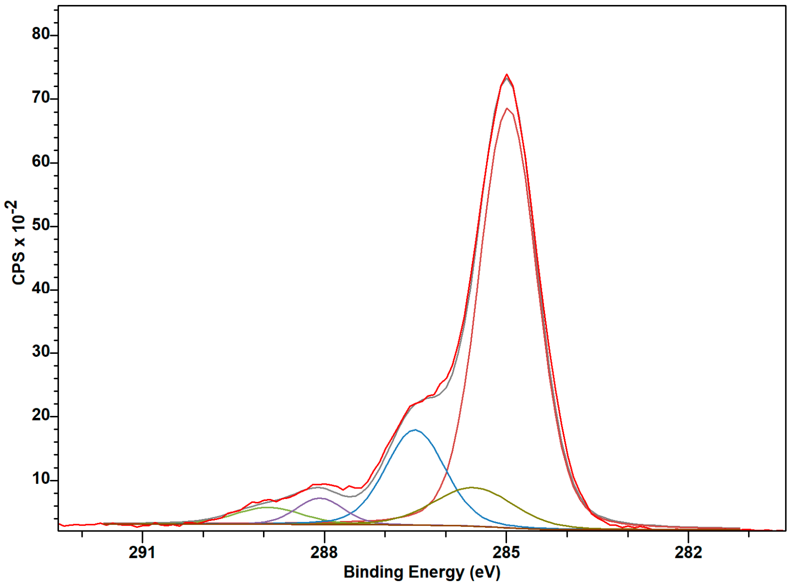

K (S11)

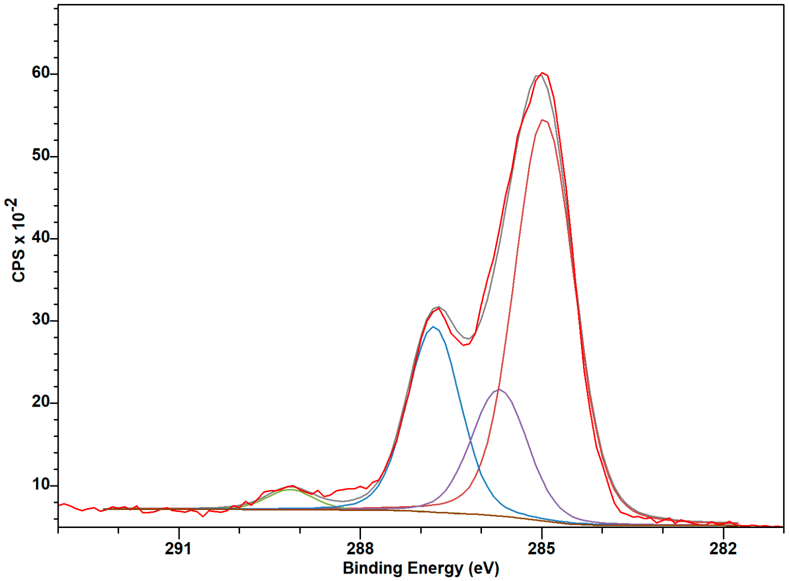

Supplemental Figure S5. High resolution X-ray photoelectron spectroscopy (XPS) spectra and decomposition of the carbon region (C1s) for the catheter samples S1 to S11 (A to K) in counts per unit (CPS) at a specified binding energy [electron volts (eV)]. The carbon decomposed into 4 peaks, ranging from 284.6 eV to 289 eV. The area of each decomposed peak matched the carbon percentage during different chemical bonding environments. Each peak was situated at 285.6 eV and 286.5 eV, which corresponded to carbon adjacent to nitrogen. The increase observed in these components was indicative of organic debris deposition on reused catheter surfaces. The amount of deposition on

intralaminar surfaces also differed from extraluminal surfaces [S4 vs S5 (images D, E); S6 vs S7 (images F, G); S8 vs S9 (images H, I); S10 vs S11 (images J, K)]. For Participant 6, composition percentage for carbon bonded to nitrogen (C1s at 285.6 eV and 286.5 eV) was relatively higher for the 30-day sample (S10, S11) than the 3-day sample (S8, S9) on both the intralaminar (S10 vs S8) and extraluminal surfaces (S11 vs S9) indicating reuse duration influenced the amount of organic debris and biofilm deposition.

Supplemental Table S1. Individual participant demographics and injury characteristics

Participant	Sex	Age*	Height (cm)	Weight (kg)	BMI (kg/m^2)	NLI	AIS	TSI (years)*	Catheter Type	Catheter Brand	Catheter Size (Fr)	Catheter Reuse (years)
1	F	48-53	180	55	16.9	C6	A	25-30	NH-PVC	Hollister (Apogee IC)	14	26
2	M	55-60	177	73	23.1	L2	D	15-20	NH-PVC	Med Rx	14	18
3	M	51-56	180	84	25.9	C6	C	25-30	NH-PVC	Med Rx	14	28
4	M	66-71	173	67	22.4	T12	D	35-40	NH-PVC	Coloplast	16	25
5	F	77-82	180	57	17.6	T12	D	40-45	NH non-latex	Amiciplus	12	42
6	F	58-63	170	68	23.5	T8	A	40-45	NH silicon	Coloplast	14	44
7	M	54-59	170	57	19.7	T9	A	30-35	H	Manfred Sauer (IQ)	14	5
8	M	70-75	175	93	30.3	T12	A	50-55	H	Coloplast (SpeediCath)	14	10
9	F	66-71	165	107	39.3	T11	D	40-45	NH-PVC	ConvaTec (Gentle Cath)	14	41
10	F	51-56	165	58	21.3	T5	A	35-40	NH-PVC	ConvaTec (Gentle Cath)	14	33

*Note: To protect the identity of the study participants, a 5-year time range is provided for information such as age and time post injury

Abbreviations: AIS = American Spinal Injury Association Impairment Scale, C = Cervical, F = Female, Fr = French, H = Hydrophilic Catheter, IC = Intermittent Catheter, L = Lumbar, M = Male, NH = Non-Hydrophilic Catheter, NH-PVC = Non-Hydrophilic Polyvinyl Chloride Catheter, NLI = Neurological Level of Injury, TSI = Time Since Injury, T = Thoracic

Supplemental Table S2. Semi-structured survey on intermittent catheterization

Item	Participant									
	1	2	3	4	5	6	7	8	9	10
Item 1	26 years	18 years	28 years	25+ years	42 years	44 years	25 years	10+ years	41 years	33 years
Item 2	4	4	5	4	6	6	6	3	9	5
Item 3	3	6	4	3	5	4	4	1	2	4
Item 4	6	10	7	8	9	10	8	5	10	6
Item 5	No bathroom access, visiting friends, delaying the need to go	Bladder infection	If drinking less, away from home or sick in bed, I catheterize less	Amount of fluid consumed	Travel - especially on buses or planes	If I don't drink and sit still, then I don't catheterize as much	If I'm busy or sick (e.g., UTI, flu, or cold) then I drink less	Drinking less or medications	Wearing an indwelling catheter and leg bag	Time spent going out, using public toilets (access, cleanliness)
Item 6	Lots of fluid	Drinking more coffee	Hot outside, working out and hydrating, drinking coffee	Having a UTI	Number of times per day I'm away from home. Forgetting when I last catheterized	If I'm active and drink a lot, I catheterize more	Drinking more, less busy, and being mindful	Drinking more	Drinking more	Having a UTI, drinking more fluids, avoiding leaks between catheterizations
Item 7	Non-hydrophilic PVC	Non-hydrophilic PVC	Non-hydrophilic PVC	Non-hydrophilic PVC	Non-hydrophilic, non-latex	Non-hydrophilic silicon	Hydrophilic	Hydrophilic (SpeediCath)	Non-hydrophilic PVC (Gentle Cath)	Non-hydrophilic PVC (Gentle Cath)
Item 8	Hollister Apogee IC	Med Rx	Med Rx	Coloplast	Amiciplus	Coloplast	IQ Cath (Manfred Sauer)	Coloplast	ConvaTec	ConvaTec
Item 9	14Fr	14Fr	14Fr	16Fr	12Fr	14Fr	14Fr	14Fr	14Fr	14Fr
Item 10	Straight tip	Straight tip	Straight tip	Straight tip	Straight tip	Straight tip	unidirectional ball tip	Straight tip	Straight tip	Straight tip
Item 11	No	No	No	No	No	No	Yes	No	No	Yes
Item 12	No	No	Yes	Yes	Yes	No	Yes	No	No	No
Item 13	Yes	Yes	Yes	Yes	Yes	Yes	Yes	Yes	Yes	Yes
Item 14	Convenience, difficulty changing	Environmental impact and convenience	Cost and I felt it could be cleaned enough for reuse	Cost	Trouble reordering and submitting reimbursement claims	Inconvenience and environmental impact	Cost (after I lost disability benefits) and environmental impact	Cost	Cost	Cost (main reason) and environmental impact
Item 15	26 years	18 years	27 years	25 years	42 years	44 years	5 years	10 years	41 years	30 years
Item 16	3 days	1 time	1 time	3 weeks	6 weeks	4 weeks	1 day	1 time	1 day	5 times (1 day)
Item 17	2 weeks	2 weeks	5 days	1 month	2 months	6 months	3 months	5 days	12 months	5 times (1 day)
Item 18	3	6	4	3	5	4	4	1	2	4
Item 19	6	10	8	8	9	10	8	5	10	6
Item 20	Rinsing with water, if out in public use soap	I don't use any cleaning method. I keep my catheters in a jar at home and carry them in my bag when I'm out.	Hot tap water	Outside of home, I first wipe with a sterile pad. After use I flush with tap water and wipe w/sterile pad before storing in original packaging	Rinse in cold followed by hot water. Inject liquid soap and allow to drain. Place catheter tip end down in tall jar containing mild soap solution. Leave 12hrs. Rinse and shake out excess water. Stand tip end up until dry	I store it in hydrogen peroxide.	Wash with water and rinse with 70% isopropyl alcohol, I let it air dry in a paper towel cardboard roller or in a Ziploc bag. When the hydrophilic coating has worn away, I use lubricant	Soap and very hot water	Put used catheters in washing machine with liquid bleach. Then soak them in hot water and vinegar. Then wrap it in a towel and put in the bathroom.	Wash it with soap and water, place it in paper towel, roll it up and put it in a bathroom drawer
Item 21	Myself	N/A	Thought of it on my own after I had to start	Determined this on my own	About 2 years ago applying all the tips I have picked up over	I decided to try it, because the solution recommended	I've always done it this way. I developed this routine on my	Urologist	In 1983, medical person told me to clean them. I used to use	Developed it on my own

			purchasing them 27 years ago		the years from nurses and a pamphlet given to me in 1980.	(Betadine) stained my clothes.	own about 5 years ago		sanitizer solution (Betadine)	
Item 22	No	No	No	Yes	No	No	No	Yes	No	No
Item 23	N/A	N/A	N/A	6-10 years after SCI	N/A	N/A	N/A	More than 10 years after SCI	N/A	N/A
Item 24	No	No	No	No	No	No	No	No	No	No
Item 25	N/A	N/A	N/A	N/A	N/A	N/A	N/A	N/A	N/A	N/A
Item 26	No	No	No	No	Yes	Yes	No	No	No	No
Item 27	N/A	N/A	N/A	N/A	One or more times per year	One or more times per year	N/A	N/A	N/A	N/A
Item 28	Yes	No	Yes	Yes	Yes	Yes	Yes	Yes	Yes	Yes
Item 29	One or more times per year	N/A	One or more times per month	One or more times per year	One or more times per month	One or more times per month	One or more times per year	One or more times per year	One or more times per week	One or more times per week
Item 30	No	No	Yes	Yes	No	Yes	Yes	Yes	No	Yes
Item 31	N/A	N/A	One or more times per year	Less than once per year	N/A	Less than once per year	One or more times per year	One or more times per year	N/A	One or more times per year
Item 32	Yes	Yes	Yes	Yes	Yes	Yes	Yes	Yes	Yes	Yes
Item 33	Less than once per year	Less than once per year	One or more times per year	One or more times per year	One or more times per year	Less than once per year	Less than once per year	One or more times per year	Less than once per year	One or more times per year
Item 34	June 2022	2015	December 2020	September 2022	07/07/2020	March 2020	Oct.-Nov. 2018	May 2022	2021	July 2022
Item 35	Only use antibiotics when necessary, last time over 20 years ago, now just drink lots of water and catheter much more frequently	If I feel it coming on, I'll change to a new catheter and see a doctor to get an antibiotic	I used to get UTI's every 2-3 months. I started increasing my water intake from 1-2L per day and haven't had a UTI since	UTI was likely caused by undrained urine in the bladder diverticulum	I had 3-4 UTIs per year. Almost always *E. coli*. By 2018 it was resistant to all antibiotics my doctor would prescribe. Started on Chinese herbs. Thought after 2 years of use I could do without. Soon got a UTI again and returned to herbs	I'd get a fever. It's hard to diagnose UTIs. They can take weeks to resolve	I had a resistant strain and needed intravenous antibiotics	Spasms, leaking and fever	I got a UTI after wearing a leg bag for serval days	Feeling uncomfortable, with body aches. I would use antibiotics and drink more fluids
Item 36	No	No	No	Yes - Ciprofloxacin	Yes - Mixture of Chinese herbs, Once per day	No	No	Yes – Whatever my urologist prescribes	Yes - Cranberry pills 1 x day	Yes - Nitrofurantoin 100mg/ night
Item 37	N/A	No	No	No	N/A	N/A	No	No	N/A	N/A
Item 38	N/A	N/A	N/A	N/A	N/A	N/A	N/A	N/A	N/A	N/A
Item 39	N/A	No	No	No	N/A	N/A	No	No	N/A	N/A
Item 40	N/A	N/A	N/A	N/A	N/A	N/A	N/A	N/A	N/A	N/A
Item 41	N/A	No	No	No	N/A	N/A	No	No	N/A	N/A
Item 42	N/A	N/A	N/A	N/A	N/A	N/A	N/A	N/A	N/A	N/A
Item 43	No	N/A	N/A	N/A	No	No	N/A	N/A	No	No
Item 44	N/A	N/A	N/A	N/A	N/A	N/A	N/A	N/A	N/A	N/A

Item 1: How long have you been using intermittent catheterization (in years)?
Item 2: Specify the average number of catheterizations you perform per day.
Item 3: Minimum number of catheterizations per day
Item 4: Maximum number of catheterizations per day
Item 5: Please describe any health-related and/ or lifestyle factors that would decrease the number of daily catheterizations you perform.
Item 6: Please describe any health-related and/ or lifestyle factors that would increase the number of daily catheterizations you perform.
Item 7: What type of catheter do you use?
Item 8: What brand of catheter are you currently using?

Item 9: What catheter size do you use most of the time (size in French/ Charrier)?
Item 10: What is the shape of the catheter tip?
Item 11: Do you clean your genital area with an antiseptic towelette or wipe before catheterizing?
Item 12: Do you put any lubricant on the catheter before catheterizing?
Item 13: Do you reuse your catheter?
Item 14: What are your reasons for reusing?
Item 15: How long have you been reusing your catheters (number of years)?
Item 16: What is the shortest amount of time you have ever reused the same catheter (please specify the number of days, weeks or months)?
Item 17: What is the longest amount of time you have ever reused the same catheter (please specify the number of days, weeks or months)?
Item 18: What is the minimum number of times per day that you reuse the same catheter?
Item 19: What is the maximum number of times per day that you reuse the same catheter?
Item 20: What methods (if any) do you use to clean/ prepare your catheter for reuse?
Item 21: When, where, and how did you learn this cleaning/ preparation method?
Item 22: Have you had any urethral injury(ies) as a result of catheterization after your SCI?
Item 23: During which period(s) did you experience urethral injury(ies) due to catheterization?
Item 24: Have you had any urethral injury(ies) as a result of catheterization by medical personnel?
Item 25: During which period(s) did you experience urethral injury(ies) due to catheterization by medical personnel?
Item 26: Do you ever experience pain in the urethra during catheterization?
Item 27: How often do you experience pain in the urethra during catheterization?
Item 28: Do you sometimes experience difficulties inserting the catheter into the urethra?
Item 29: How often do you experience difficulties inserting the catheter?
Item 30: Do you ever notice blood on the catheter after withdrawal from the urethra?
Item 31: How often do you notice blood on the catheter after withdrawal from the urethra?
Item 32: Have you ever had a urinary tract infection (UTI)?
Item 33: How frequently do you get UTIs?
Item 34: What was the approximate date of the last UTI you had?
Item 35: Please describe any additional experiences with regards to having and/or resolving UTIs?
Item 36: Do you use any prophylactic measures (e.g. antibiotics) to avoid getting UTIs? If yes, please specify what you use and how often you use it.
Item 37: (Males only) Have you ever experienced any inflammation in the testicles?
Item 38: (Males only) During which period(s) did you experience inflammation in the testicles?
Item 39: (Males only) Have you ever experienced any inflammation in the epididymis?
Item 40: (Males only) During which period(s) did you experience inflammation in the epididymis?
Item 41: (Males only) Have you ever experienced any inflammation in the prostate?
Item 42: (Males only) During which period(s) did you experience inflammation in the prostate?
Item 43: (Females only) Have you ever experienced any pelvic inflammatory conditions?
Item 44: (Females only) During which period(s) did you experience pelvic inflammation?

Abbreviations: Fr = French, PVC = Polyvinyl Chloride, SCI = Spinal Cord Injury, UTI = Urinary Tract Infection

Supplemental Table S3. Catheter swab gram smear and culture analysis

Participant	Day	Swab #	Collection Time	Gram Stain	Culture	Susceptibilities
1	1	1	8:00-12:00	No cells seen	No growth after 48hrs	
		2	12:00-16:00	No cells seen	No growth after 48hrs	
		3	16:00-20:00	(1) Rare epithelial cells<br>(2) Rare gram positive bacilli<br>(3) Rare gram positive cocci<br>(4) Rare gram negative bacilli<br>(5) No neutrophils seen	(1) Mixed flora†	
	2	4	8:00-12:00	(1) Rare epithelial cells<br>(2) Rare neutrophils	(1) *Escherichia coli* (scant growth)<br>(2) Mixed flora† (scant growth)	(1) (S)Ampicillin; (S)Tetracyclin; (S)Gentamicin; (S)Trimethoprim-Sulfamethoxazole; (S)Ciprofloxacin; (S)Ceftriaxone
		5	12:00-16:00	(1) Rare epithelial cells<br>(2) No neutrophils seen	(1) *Acinetbacter species* (scant growth)	(1) (S)Tetracyclin; (S)Gentamicin; (S)Trimethoprim-Sulfamethoxazole; (S)Ciprofloxacin; (S)Ceftriaxone
		6	16:00-20:00	(1) Rare epithelial cells<br>(2) No neutrophils seen	(1) Skin flora†	
	3	7	8:00-12:00	No cells seen	No growth after 48hrs	
		8	12:00-16:00	No cells seen	(1) Skin flora†	
		9	16:00-20:00	(1) Rare gram positive cocci<br>(2) Rare gram negative bacilli<br>(3) No cells seen	(1) *Escherichia coli* (moderate growth)<br>(2) Skin flora† (light growth)	(1) (S)Ampicillin; (S)Tetracyclin; (S)Gentamicin; (S)Trimethoprim-Sulfamethoxazole; (S)Ciprofloxacin; (S)Ceftriaxone
2	1	1	8:00-12:00	No cells seen	No growth after 48hrs	
		2	12:00-16:00	(1) Few gram negative bacilli<br>(2) No cells seen	(1) Mixed flora†	
		3	16:00-20:00	(1) Rare gram negative bacilli<br>(2) No cells seen	(1) Mixed flora†	
	2	4	8:00-12:00	(1) Rare epithelial cells<br>(2) Rare gram positive cocci<br>(3) Rare gram positive bacilli<br>(4) Rare gram negative bacilli<br>(5) No neutrophils seen	(1) Mixed flora†	
		5	12:00-16:00	(1) Rare epithelial cells<br>(2) Rare neutrophils<br>(3) Rare gram positive cocci<br>(4) Rare gram positive bacilli<br>(5) Rare gram negative bacilli	(1) Mixed flora†	
		6	16:00-20:00	(1) Rare epithelial cells<br>(2) Rare neutrophils<br>(3) Rare gram positive cocci<br>(4) Rare gram negative bacilli	(1) Mixed flora†	
	3	7	8:00-12:00	No cells seen	(1) Mixed flora†	
		8	12:00-16:00	No cells seen	(1) Mixed flora†	
		9	16:00-20:00	(1) Rare gram positive bacilli<br>(2) No cells seen	(1) Mixed flora†	
3	1	1	8:00-12:00	(1) Rare red blood cells present<br>(2) No neutrophils seen	(1) Skin flora†	
		2	12:00-16:00	No cells seen	No growth after 48hrs	
		3	16:00-20:00	No cells seen	No growth after 48hrs	

	2	4	8:00-12:00	No cells seen	(1) Skin flora†	
		5	12:00-16:00	No cells seen	No growth after 48hrs	
		6	16:00-20:00	No cells seen	(1) *Staphylococcus aureus* (light growth)<br>(2) *Staphylococcus lugdunensis* (scant growth)	(1) (S)Tetracyclin; (S)Trimethoprim-Sulfamethoxazole; (**R**)Erythromycin; (S)Cloxacillin; (S)Cephlothin / Cephalexin; (**R**)Clindamycin<br>(2) (S)Tetracyclin; (S)Trimethoprim-Sulfamethoxazole; (S)Erythromycin; (S)Cloxacillin; (S)Cephlothin / Cephalexin; (S)Clindamycin
	3	7	8:00-12:00	No cells seen	(1) Skin flora†	
		8	12:00-16:00	No cells seen	(1) Skin flora†	
		9	16:00-20:00	No cells seen	(1) Skin flora†	
4	1	1	8:00-12:00	(1) Rare gram positive bacilli<br>(2) No cells seen	(1) Skin flora†	
		2	12:00-16:00	(1) Rare gram positive bacilli<br>(2) No cells seen	No growth after 48hrs	
		3	16:00-20:00	(1) Rare gram positive bacilli<br>(2) No cells seen	No growth after 48hrs	
	2	4	8:00-12:00	No cells seen	No growth after 48hrs	
		5	12:00-16:00	No cells seen	No growth after 48hrs	
		6	16:00-20:00	No cells seen	No growth after 48hrs	
	3	7	8:00-12:00	No cells seen	No growth after 48hrs	
		8	12:00-16:00	No cells seen	No growth after 48hrs	
		9	16:00-20:00	(1) Rare epithelial cells<br>(2) No neutrophils seen	No growth after 48hrs	
5	1	1	8:00-12:00	No cells seen	No growth after 48hrs	
		2	12:00-16:00	No cells seen	No growth after 48hrs	
		3	16:00-20:00	No cells seen	No growth after 48hrs	
	2	4	8:00-12:00	(1) Rare epithelial cells<br>(2) Rare neutrophils	(1) Skin flora†	
		5	12:00-16:00	(1) Rare epithelial cells<br>(2) Rare neutrophils	(1) Skin flora†	
		6	16:00-20:00	(1) Rare epithelial cells<br>(2) Rare neutrophils	No growth after 48hrs	
	3	7	8:00-12:00	No cells seen	(1) Skin flora†	
		8	12:00-16:00	No cells seen	No growth after 48hrs	
		9	16:00-20:00	(1) Rare epithelial cells<br>(2) Rare gram positive bacilli<br>(3) No neutrophils seen	(1) Skin flora†	
6	1	1	8:00-12:00	(1) Few gram positive bacilli<br>(2) No cells seen	No growth after 48hrs	
		2	12:00-16:00	(1) Rare gram positive bacilli<br>(2) No cells seen	No growth after 48hrs	
		3	16:00-20:00	No cells seen	No growth after 48hrs	
	2	4	8:00-12:00	No cells seen	No growth after 48hrs	
		5	12:00-16:00	No cells seen	No growth after 48hrs	
		6	16:00-20:00	(1) Rare epithelial cells<br>(2) No neutrophils seen	No growth after 48hrs	
	3	7	8:00-12:00	No cells seen	No growth after 48hrs	
		8	12:00-16:00	No cells seen	No growth after 48hrs	
		9	16:00-20:00	(1) Few epithelial cells	No growth after 48hrs	

				(2) No neutrophils seen		
7	1	1	8:00-12:00	No cells seen	No growth after 48hrs	
		2	12:00-16:00	No cells seen	(1) Mixed flora†	
		3	16:00-20:00	No cells seen	No growth after 48hrs	
	2	4	8:00-12:00	(1) Rare epithelial cells<br>(2) Rare neutrophils<br>(3) Rare gram positive cocci<br>(4) Rare gram negative bacilli	(1) *Klebsiella pneumoniae* (light growth)<br>(2) Skin flora† (scant growth)	(1) (**R**)Ampicillin; (S)Tetracyclin; (S)Gentamicin; (S)Trimethoprim-Sulfamethoxazole; (S)Ciprofloxacin; (S)Ceftriaxone; (S)Cefixime;
		5	12:00-16:00	(1) Rare epithelial cells<br>(2) Rare neutrophils	(1) Mixed flora†	
		6	16:00-20:00	No cells seen	(1) Mixed flora†	
	3	7	8:00-12:00	(1) Rare gram positive bacilli<br>(2) No cells seen	(1) Mixed flora†	
		8	12:00-16:00	No cells seen	(1) Mixed flora†	
		9	16:00-20:00	No cells seen	No growth after 48hrs	
8	1	1	8:00-12:00	No cells seen	No growth after 48hrs	
		2	12:00-16:00	No cells seen	No growth after 48hrs	
		3	16:00-20:00	No cells seen	(1) Skin flora†	
	2	4	8:00-12:00	(1) Rare epithelial cells<br>(2) No neutrophils seen	(1) Skin flora†	
		5	12:00-16:00	(1) Rare epithelial cells<br>(2) Rare neutrophils	(1) Skin flora†	
		6	16:00-20:00	(1) Rare epithelial cells<br>(2) No neutrophils seen	(1) Skin flora†	
	3	7	8:00-12:00	No cells seen	(1) Skin flora†	
		8	12:00-16:00	(1) Rare gram positive bacilli<br>(2) No cells seen	(1) Skin flora†	
		9	16:00-20:00	No cells seen	(1) Skin flora†	
9	1	1	8:00-12:00	No cells seen	No growth after 48hrs	
		2	12:00-16:00	No cells seen	No growth after 48hrs	
		3	16:00-20:00	No cells seen	No growth after 48hrs	
	2	4	8:00-12:00	(1) Rare epithelial cells<br>(2) Rare gram positive cocci<br>(3) No neutrophils seen	(1) Skin flora†	
		5	12:00-16:00	(1) Rare epithelial cells<br>(2) Rare neutrophils	No growth after 48hrs	
		6	16:00-20:00	(1) Rare epithelial cells<br>(2) No neutrophils seen	No growth after 48hrs	
	3	7	8:00-12:00	No cells seen	No growth after 48hrs	
		8	12:00-16:00	No cells seen	No growth after 48hrs	
		9	16:00-20:00	No cells seen	No growth after 48hrs	
10	1	1	8:00-12:00	No cells seen	No growth after 48hrs	
		2	12:00-16:00	No cells seen	(1) Skin flora†	
		3	16:00-20:00	(1) Rare gram positive bacilli<br>(2) No cells seen	(1) Skin flora†	
	2	4	8:00-12:00	(1) Few epithelial cells<br>(2) No neutrophils seen	(1) Skin flora†	
		5	12:00-16:00	(1) No cells seen	(1) *Escherichia coli* (scant growth)	(1) (S)Ampicillin; (S)Tetracyclin; (S)Gentamicin; (S)Trimethoprim-Sulfamethoxazole; (S)Ciprofloxacin; (S)Ceftriaxone

		6	16:00-20:00	(1) No cells seen	(1) *Escherichia coli* (scant growth)	(1) (S)Ampicillin; (S)Tetracyclin; (S)Gentamicin; (S)Trimethoprim-Sulfamethoxazole; (S)Ciprofloxacin; (S)Ceftriaxone
	3	7	8:00-12:00	(1) Few epithelial cells<br>(2) No neutrophils seen	(1) Mixed flora†	
		8	12:00-16:00	No cells seen	(1) *Escherichia coli* (scant growth)	(1) (S)Ampicillin; (S)Tetracyclin; (S)Gentamicin; (S)Trimethoprim-Sulfamethoxazole; (S)Ciprofloxacin; (S)Ceftriaxone
		9	16:00-20:00	(1) Rare epithelial cells<br>(2) No neutrophils seen	(1) Skin flora†	

†No further analyses conducted

Abbreviations: AMP = Ampicillin; CEF = Cefazolin; CFM = Cefixime; CPFX = Ciprofloxacin; CRO = Ceftriaxone; FOS = Fosfomycin; GEN = Gentamicin; I = Intermediate Resistance; NF = Nitrofurantoin; R = Resistant; S = Susceptible; TCN = Tetracycline; TMP-SMX = Trimethoprim-sulfamethoxazole

Supplemental Table S4. Secondary catheter culture analysis

Participant	Catheter Sample	Catheter Segment	Culture (M CFU/L)
Participant 3	Pristine control	First 1.5cm of the catheter tip	No growth
Participant 4	Pristine control	First 1.5cm of the catheter tip	No growth
Participant 6	Pristine control	First 1.5cm of the catheter tip	No growth
Participant 3	Reused (3 days)	First 1.5cm of the catheter tip	40 M CFU/L
Participant 4	Reused (3 days)	First 1.5cm of the catheter tip	No growth
Participant 6	Reused (3 days)	First 1.5cm of the catheter tip	No growth
Participant 6	Reused (30 days)	First 1.5cm of the catheter tip	No growth

Abbreviations: M CFU/L = Million Colony Forming Units per Liter

Supplemental Table S5. Urine chemistry analysis

Participant	Urine Sample	Color	Appearance	pH (5.0-8.0)	Specific Gravity (1.003-1.035)	Protein (g/L)	Glucose (mmol/L)	Ketones (mmol/L)	Hemoglobin (mg/L)	Nitrite (-)/(+)	Leukocytes (WBC/uL)
1	1 (Baseline)	Yellow	Clear	6.5	1.010	<0.3	<2.8	<0.5	<0.3	-	<25
	2 (Day 1)	Yellow	Clear	6.5	1.010	<0.3	<2.8	<0.5	<0.3	-	<25
	3 (Day 2)	Yellow	**Cloudy**	6	1.010	<0.3	<2.8	<0.5	<0.3	-	<25
	4 (Day 3)	Yellow	Clear	5.5	1.010	<0.3	<2.8	<0.5	<0.3	+	**70**
	5 (Follow-Up)	Yellow	**Cloudy**	6.5	1.010	<0.3	<2.8	**1.5**	<0.3	+	<25
2	1 (Baseline)	Yellow	Clear	8	1.010	<0.3	<2.8	<0.5	<0.3	-	**125**
	2 (Day 1)	Yellow	Clear	8	1.010	<0.3	<2.8	<0.5	<0.3	-	**125**
	3 (Day 2)	Yellow	Clear	6.5	**≤1.005**	<0.3	<2.8	<0.5	**Trace**	+	**500**
	4 (Day 3)	Yellow	Clear	8	**≤1.005**	<0.3	<2.8	<0.5	<0.3	+	**500**
	5 (Follow-Up)	Yellow	Clear	5.5	1.015	<0.3	<2.8	<0.5	<0.3	-	**70**
3	1 (Baseline)	**Dark Yellow**	**Cloudy**	6	1.020	<0.3	<2.8	<0.5	**25**	-	**500**
	2 (Day 1)	**Dark Yellow**	**Turbid**	7.5	1.020	<0.3	<2.8	<0.5	**25**	+	**500**
	3 (Day 2)	**Dark Yellow**	**Turbid**	6.5	1.025	<0.3	<2.8	<0.5	**25**	-	**500**
	4 (Day 3)	Yellow	Clear	5.5	**≤1.005**	<0.3	<2.8	<0.5	**Trace**	-	**70**
	5 (Follow-Up)	Yellow	**Cloudy**	5.5	1.020	<0.3	<2.8	<0.5	**80**	-	**500**
4	1 (Baseline)	Yellow	**Cloudy**	7	1.015	<0.3	<2.8	<0.5	<0.3	-	**125**
	2 (Day 1)	Yellow	**Cloudy**	6.5	1.015	<0.3	<2.8	<0.5	<0.3	-	**125**
	3 (Day 2)	Yellow	Clear	6	1.020	**0.3**	<2.8	<0.5	<0.3	-	**500**
	4 (Day 3)	Yellow	**Turbid**	6.5	1.020	<0.3	<2.8	<0.5	**25**	+	**500**
	5 (Follow-Up)	Yellow	**Turbid**	7.5	1.015	<0.3	<2.8	<0.5	**Trace**	+	**500**
5	1 (Baseline)	Yellow	Clear	7.5	**≤1.005**	<0.3	<2.8	<0.5	<0.3	-	**125**
	2 (Day 1)	Yellow	Clear	7.5	**≤1.005**	<0.3	<2.8	<0.5	<0.3	-	**125**
	3 (Day 2)	Yellow	**Cloudy**	5	1.015	<0.3	<2.8	<0.5	<0.3	+	**70**
	4 (Day 3)	Yellow	Clear	8	**≤1.005**	<0.3	<2.8	<0.5	**25**	-	<25
	5 (Follow-Up)	Yellow	Clear	6.5	1.010	<0.3	<2.8	<0.5	**Trace**	-	<25
6	1 (Baseline)	Yellow	Clear	7.5	1.015	<0.3	<2.8	<0.5	<0.3	-	**125**
	2 (Day 1)	Yellow	Clear	5	1.010	<0.3	<2.8	<0.5	<0.3	+	**125**
	3 (Day 2)	Yellow	Clear	5	1.015	<0.3	<2.8	<0.5	<0.3	+	**125**
	4 (Day 3)	Yellow	**Cloudy**	6.5	1.015	<0.3	<2.8	<0.5	<0.3	+	**125**
	5 (Follow-Up)	Yellow	Clear	5	1.015	<0.3	<2.8	<0.5	<0.3	+	**70**
7	1 (Baseline)	Yellow	Clear	6.5	**≤1.005**	<0.3	<2.8	<0.5	<0.3	-	**500**
	2 (Day 1)	Yellow	Clear	6.5	**≤1.005**	<0.3	<2.8	<0.5	<0.3	-	**125**
	3 (Day 2)	Yellow	Clear	6.5	**≤1.005**	<0.3	<2.8	<0.5	<0.3	-	**125**
	4 (Day 3)	Yellow	Clear	6.5	**≤1.005**	<0.3	<2.8	<0.5	<0.3	-	**125**
	5 (Follow-Up)	Yellow	Clear	7	**≤1.005**	<0.3	<2.8	<0.5	<0.3	-	**125**
8	1 (Baseline)	Yellow	Clear	5.5	1.015	<0.3	<2.8	<0.5	<0.3	+	**500**
	2 (Day 1)	Yellow	Clear	5.5	1.015	<0.3	<2.8	<0.5	<0.3	+	**500**
	3 (Day 2)	Yellow	Clear	5.5	1.010	<0.3	<2.8	<0.5	<0.3	-	**70**
	4 (Day 3)	Yellow	Clear	5.5	1.025	<0.3	<2.8	<0.5	<0.3	+	**125**
	5 (Follow-Up)	Yellow	Clear	6	1.020	<0.3	<2.8	<0.5	<0.3	+	**125**
9	1 (Baseline)	**Dark Yellow**	**Cloudy**	7.5	1.015	<0.3	<2.8	<0.5	<0.3	-	<25
	2 (Day 1)	Yellow	Clear	5.5	1.025	<0.3	<2.8	<0.5	<0.3	-	**70**
	3 (Day 2)	Yellow	**Cloudy**	6.5	1.025	<0.3	<2.8	<0.5	<0.3	+	<25
	4 (Day 3)	Yellow	Clear	6	1.025	<0.3	<2.8	<0.5	<0.3	-	<25
	5 (Follow-Up)	Yellow	Clear	6	1.020	<0.3	<2.8	<0.5	<0.3	+	<25

10	1 (Baseline)	Yellow	**Turbid**	**≥9.0**	1.015	0.3	<2.8	<0.5	**Trace**	+	**500**
	2 (Day 1)	Yellow	Clear	7	1.010	<0.3	<2.8	<0.5	<0.3	-	**125**
	3 (Day 2)	Yellow	Clear	7.5	1.015	<0.3	<2.8	<0.5	<0.3	-	**70**
	4 (Day 3)	Yellow	**Cloudy**	7.5	1.015	<0.3	<2.8	<0.5	<0.3	+	**125**
	5 (Follow-Up)	Yellow	**Cloudy**	5.5	1.020	<0.3	<2.8	<0.5	**25**	+	**500**

Positive analysis results are labelled in **bold**

Abbreviations: WBC = White Blood Cell

Supplemental Table S6. Urine culture analysis

Participant	Urine Sample	Culture (M CFU/L)	Antibiotic Susceptibility											
			**AMP**	**TCN**	**GEN**	**NF**	**TMP-SMX**	**CPFX**	**VAN**	**CRO**	**CFM**	**FOS**	**CEF**	**Other(s)**
1	1 (Baseline)	(1) *Escherichia coli* (>100M); (2) *Enterococcus faecalis* (>100M)	(1)S, (2)S	(1)S, (2)S	(1)S	(1)S, (2)S	(1)S	(1)S, (2)S	(2) S	(1)S	(1)S	(1)S	(1)S	-
	2 (Day 1)	1) *Escherichia coli* (>100M); (2) *Enterococcus faecalis* (>100M)	(1)S, (2)S	(1)S, (2)S	(1)S	(1)S, (2)S	(1)S	(1)S, (2)S	(2) S	(1)S	(1)S	(1)S	(1)S	-
	3 (Day 2)	1) *Escherichia coli* (>100M); (2) *Enterococcus faecalis* (>100M)	(1)S, (2)S	(1)S, (2)S	(1)S	(1)S, (2)S	(1)S	(1)S, (2)S	(2) S	(1)S	(1)S	(1)S	(1)S	-
	4 (Day 3)	1) *Escherichia coli* (>100M); (2) *Enterococcus faecalis* (>100M)	(1)S, (2)S	(1)S, (2)S	(1)S	(1)S, (2)S	(1)S	(1)S, (2)S	(2) S	(1)S	(1)S	(1)S	(1)S	-
	5 (Follow-Up)	1) *Escherichia coli* (>100M); (2) *Enterococcus faecalis* (>100M)	(1)S, (2)S	(1)S, (2)S	(1)S	(1)S, (2)S	(1)S	(1)S, (2)S	(2) S	(1)S	(1)S	(1)S	(1)S	-
2	1 (Baseline)	(1) *Escherichia coli* (>100M); (2) *Klebsiella pneumoniae* (>100M)	(1)S, (2)**R**	(1)S, (2)S	(1)S, (2)S	(1)S, (2)**I**	(1)S, (2)S	(1)**R**, (2)S	-	(1)S, (2)S	(1)S, (2)S	(1)S	(1)S, (2)S	-
	2 (Day 1)	(1) *Escherichia coli* (>100M); (2) *Klebsiella pneumoniae* (>100M)	(1)S, (2)**R**	(1)S, (2)S	(1)S, (2)S	(1)S, (2)**I**	(1)S, (2)S	(1)**R**, (2)S	-	(1)S, (2)S	(1)S, (2)S	(1)S	(1)S, (2)S	-
	3 (Day 2)	(1) *Escherichia coli* (>100M); (2) *Klebsiella pneumoniae* (>100M)	(1)S, (2)**R**	(1)S, (2)S	(1)S, (2)S	(1)S, (2)**I**	(1)S, (2)**R**	(1)**R**, (2)S	-	(1)S, (2)S	(1)S, (2)S	(1)S	(1)S, (2)S	-
	4 (Day 3)	(1) *Escherichia coli* (>100M); (2) *Klebsiella pneumoniae* (>100M)	(1)S, (2)**R**	(1)S, (2)S	(1)S, (2)S	(1)S, (2)**I**	(1)S, (2)S	(1)**R**, (2)S	-	(1)S, (2)S	(1)S, (2)S	(1)S	(1)S, (2)S	-
	5 (Follow-Up)	(1) *Escherichia coli* (>100M); (2) *Klebsiella pneumoniae* (>100M)	(1)S, (2)**R**	(1)S, (2)S	(1)S, (2)S	(1)S, (2)**R**	(1)S, (2)**R**	(1)**R**, (2)S	-	(1)S, (2)S	(1)S, (2)S	(1)S	(1)S, (2)S	-
3	1 (Baseline)	(1) *Klebsiella pneumoniae* (>100M)	(1)**R**	(1)**R**	(1)S	(1)S	(1)S	(1)S	-	(1)S	(1)S	-	(1)S	-
	2 (Day 1)	(1) *Klebsiella pneumoniae* (>100M)	(1)**R**	(1)**R**	(1)S	(1)S	(1)S	(1)S	-	(1)S	(1)S	-	(1)S	-
	3 (Day 2)	(1) *Klebsiella pneumoniae* (>100M)	(1)**R**	(1)**R**	(1)S	(1)S	(1)S	(1)S	-	(1)S	(1)S	-	(1)S	-
	4 (Day 3)	(1) *Klebsiella pneumoniae* (>100M)	(1)**R**	(1)**R**	(1)S	(1)S	(1)S	(1)S	-	(1)S	(1)S	-	(1)S	-
	5 (Follow-Up)	(1) *Klebsiella pneumoniae* (>100M)	(1)**R**	(1)**R**	(1)S	(1)S	(1)S	(1)S	-	(1)S	(1)S	-	(1)S	-
4	1 (Baseline)	No growth	-	-	-	-	-	-	-	-	-	-	-	-
	2 (Day 1)	No growth	-	-	-	-	-	-	-	-	-	-	-	-
	3 (Day 2)	(1) *Pseudomonas aeruginosa* (10-100M)	-	-	(1)S	-	-	(1)**R**	-	-	-	-	-	(1) CTZ(S); MEM(I); PIP-TZP(S)
	4 (Day 3)	(1) *Pseudomonas aeruginosa* (>100M)	-	-	(1)S	-	-	(1)**R**	-	-	-	-	-	(1) CTZ(S); MEM(I); PIP-TZP(S)
	5 (Follow-Up)	(1) *Pseudomonas aeruginosa* (>100M)	-	-	(1)S	-	-	(1)**R**	-	-	-	-	-	(1) CTZ(S); MEM(I); PIP-TZP(S)
5	1 (Baseline)	(1) *Escherichia coli* (>100M)	(1)**R**	(1)S	(1)S	(1)S	(1)S	(1)S	-	(1)**R**	-	(1)S	-	(1) MEM(S); ERT(S)
	2 (Day 1)	(1) *Escherichia coli* (>100M)	(1)**R**	(1)S	(1)S	(1)S	(1)S	(1)S	-	(1)**R**	-	(1)S	-	(1) MEM(S); ERT(S)
	3 (Day 2)	(1) *Escherichia coli* (>100M)	(1)**R**	(1)S	(1)S	(1)S	(1)S	(1)S	-	(1)**R**	-	(1)S	-	(1) MEM(S); ERT(S)
	4 (Day 3)	(1) *Escherichia coli* (>100M)	(1)**R**	(1)S	(1)S	(1)S	(1)S	(1)S	-	(1)**R**	-	(1)S	-	(1) MEM(S); ERT(S)
	5 (Follow-Up)	(1) *Escherichia coli* (10-100M)	(1)**R**	(1)S	(1)S	(1)S	(1)S	(1)S	-	(1)S	-	(1)S	(1)S	-

6	1 (Baseline)	(1) *Escherichia coli* (>100M)	(1)S	(1)S	(1)S	(1)S	(1)S	(1)S	-	(1)S	(1)S	(1)S	(1)S	-
	2 (Day 1)	(1) *Escherichia coli* (>100M)	(1)S	(1)S	(1)S	(1)S	(1)S	(1)S	-	(1)S	(1)S	(1)S	(1)S	-
	3 (Day 2)	(1) *Escherichia coli* (>100M)	(1)S	(1)S	(1)S	(1)S	(1)S	(1)S	-	(1)S	(1)S	(1)S	(1)S	-
	4 (Day 3)	(1) *Escherichia coli* (>100M)	(1)S	(1)S	(1)S	(1)S	(1)S	(1)S	-	(1)S	(1)S	(1)S	(1)S	-
	5 (Follow-Up)	(1) *Escherichia coli* (>100M)	(1)S	(1)S	(1)S	(1)S	(1)S	(1)S	-	(1)S	(1)S	(1)S	(1)S	-
7	1 (Baseline)	(1) *Klebsiella pneumoniae* (>100M)	(1)**R**	(1)S	(1)S	(1)S	(1)S	(1)S	-	(1)S	(1)S	(1)S	(1)S	-
	2 (Day 1)	(1) *Klebsiella pneumoniae* (>100M)	(1)**R**	(1)S	(1)S	(1)S	(1)S	(1)S	-	(1)S	(1)S	(1)S	(1)S	-
	3 (Day 2)	(1) *Klebsiella pneumoniae* (>100M)	(1)**R**	(1)S	(1)S	(1)S	(1)S	(1)S	-	(1)S	(1)S	(1)S	(1)S	-
	4 (Day 3)	(1) *Klebsiella pneumoniae* (>100M)	(1)**R**	(1)S	(1)S	(1)S	(1)S	(1)S	-	(1)S	(1)S	(1)S	(1)S	-
	5 (Follow-Up)	(1) *Klebsiella pneumoniae* (>100M)	(1)**R**	(1)S	(1)S	(1)S	(1)S	(1)S	-	(1)S	(1)S	(1)S	(1)S	-
8	1 (Baseline)	(1) *Enterococcus faecalis* (>100M);<br>(2) *Acinetobacter calcoaceticus-baumannii complex* (>100M)	(1)S	(1)S,<br>(2)S	(2)S	(1)S	(2)S	(1)S,<br>(2)S	(1)S	-	-	-	-	(2) CTZ(S);<br>MEM(S);<br>TOB(S)
	2 (Day 1)	(1) *Enterococcus faecalis* (>100M);<br>(2) *Acinetobacter calcoaceticus-baumannii complex* (>100M)	(1)S	(1)S,<br>(2)S	(2)S	(1)S	(2)S	(1)S,<br>(2)S	(1)S	-	-	-	-	(2) CTZ(S);<br>MEM(S);<br>TOB(S)
	3 (Day 2)	(1) *Enterococcus faecalis* (>100M);<br>(2) *Acinetobacter calcoaceticus-baumannii complex* (>100M)	(1)S	(1)S,<br>(2)S	(2)S	(1)S	(2)S	(1)S,<br>(2)S	(1)S	-	-	-	-	(2) CTZ(S);<br>MEM(S);<br>TOB(S)
	4 (Day 3)	(1) *Enterococcus faecalis* (10-100M);<br>(2) *Acinetobacter pittii* (10-100M)	(1)S	(1)S,<br>(2)S	(2)S	(1)S	(2)S	(1)S,<br>(2)S	(1)S	-	-	-	-	(2) CTZ(S)
	5 (Follow-Up)	(1) Enterococcus faecalis (>100M)	(1)S	(1)S	-	(1)S	-	(1)S	(1)S	-	-	-	-	-
9	1 (Baseline)	(1) *Escherichia coli* (10-100M);<br>(2) *Enterococcus faecalis* (>100M)	(1)S,<br>(2)S	(1)S,<br>(2)**R**	(1)S	(1)S,<br>(2)S	(1)S	(1)S,<br>(2)S	(2)S	(1)S	(1)S	(1)S	(1)S	-
	2 (Day 1)	(1) *Escherichia coli* (>100M);<br>(2) *Enterococcus faecalis* (>100M)	(1)S,<br>(2)S	(1)S,<br>(2)**R**	(1)S	(1)S,<br>(2)S	(1)S	(1)S,<br>(2)S	(2)S	(1)S	(1)S	(1)S	(1)S	-
	3 (Day 2)	(1) *Escherichia coli* (>100M);<br>(2) *Enterococcus faecalis* (10-100M)	(1)S,<br>(2)S	(1)S,<br>(2)**R**	(1)S	(1)S,<br>(2)S	(1)S	(1)S,<br>(2)S	(2)S	(1)S	(1)S	(1)S	(1)S	-
	4 (Day 3)	(1) *Escherichia coli (*10-100M)	(1)S	(1)S	(1)S	(1)S	(1)S	(1)S	(1)S	(1)S	(1)S	(1)S	(1)S	-
	5 (Follow-Up)	(1) *Escherichia coli* (>100M)	(1)S	(1)S	(1)S	(1)S	(1)S	(1)S	(1)S	(1)S	(1)S	(1)S	(1)S	-
10	1 (Baseline)	(1) *Proteus vulgaris* (>100M)	-	(1)**R**	(1)S	(1)**R**	(1)**R**	(1)**R**	-	-	-	(1)**R**	-	(1) MEM(S);<br>ERT(S)
	2 (Day 1)	(1) *Escherichia coli* (>100M)	(1)S	(1)S	(1)S	(1)S	(1)S	(1)S	-	(1)S	(1)S	(1)**R**	(1)S	-
	3 (Day 2)	(1) *Escherichia coli (*10-100M)	(1)S	(1)S	(1)S	(1)S	(1)S	(1)S	-	(1)S	(1)S	(1)**R**	(1)S	-
	4 (Day 3)	(1) *Escherichia coli* (>100M)	(1)S	(1)S	(1)S	(1)S	(1)S	(1)S	-	(1)S	(1)S	(1)**R**	(1)S	-
	5 (Follow-Up)	(1) *Escherichia coli* (>100M)	(1)S	(1)S	(1)S	(1)S	(1)S	(1)S	-	(1)S	(1)S	(1)**R**	(1)S	-

Abbreviations: AMP = Ampicillin; CEF = Cefazolin; CFM = Cefixime; CPFX = Ciprofloxacin; CRO = Ceftriaxone; CTZ = Ceftazidime; ERT = Ertapenem; FOS = Fosfomycin; GEN = Gentamicin; I = Intermediate Resistance; M CFU/L = Million Colony Forming Units per Liter; MEM = Meropenem; NF = Nitrofurantoin; PIP-TZP = Piperacillin-Tazobactam (TZP); R = Resistant; S = Susceptible; TCN = Tetracycline; TMP-SMX = Trimethoprim-Sulfamethoxazole; TOB = Tobramycin; VAN = Vancomycin

Supplemental Appendix A. Incontinence - Quality of Life

Item 1. I worry about not being able to get to the toilet on time.
Item 2. I worry about coughing and sneezing.
Item 3. I have to be careful about standing up after sitting down.
Item 4. I worry where the toilets are in new places.
Item 5. I feel depressed.
Item 6. I don’t feel free to leave my home for long periods of time.
Item 7. I feel frustrated because my urinary incontinence (UI) prevents me from doing what I want.
Item 8. I worry about others smelling urine on me.
Item 9. Incontinence is always on my mind.
Item 10. It’s important for me to make frequent trips to the toilet.
Item 11. Because of my incontinence, it is important to plan every detail in advance.
Item 12. I worry about my incontinence getting worse as I grow older.
Item 13. I have a hard time getting a good night’s sleep.
Item 14. I worry about being embarrassed or humiliated because of my incontinence.
Item 15. My incontinence makes me feel like I’m not a healthy person.
Item 16. My UI makes me feel helpless.
Item 17. I get less enjoyment out of life because of my UI.
Item 18. I worry about wetting myself.
Item 19. I feel like I have no control over my bladder.
Item 20. I have to watch what I drink.
Item 21. My UI limits my choice of clothing.
Item 22. I worry about having sex.

All items use the following response scale:
1 = Extremely
2 = Quite a bit
3 = Moderately
4 = A little
5 = Not at all

Subscale structure:
Avoidance and limiting behavior: items 1, 2, 3, 4, 10, 11, 13, and 20
Psychosocial impacts: items 5, 6, 7, 9, 15, 16, 17, 21, and 22
Social embarrassment: items 8, 12, 14, 18 and 19

Supplemental Appendix B. Intermittent catheterization questionnaire

Section 1. Intermittent Catheterization

Item 1: How long have you been using intermittent catheterization (in years)?

____

Item 2: Specify the average number of catheterizations you perform per day.

☐ 1 ☐ 2 ☐ 3 ☐ 4 ☐ 5 ☐ 6 ☐ 7 ☐ 8 ☐ 9 ☐ 10 or more times

Item 3: Minimum number of catheterizations per day

____

Item 4: Maximum number of catheterizations per day

____

Item 5: Please describe any health-related and/ or lifestyle factors that would decrease the number of daily catheterizations you perform.

____
____
____

Item 6: Please describe any health-related and/ or lifestyle factors that would increase the number of daily catheterizations you perform.

____
____
____

Item 7: What type of catheter do you use?

☐ Hydrophilic catheter ☐ Non-hydrophilic PVC catheter ☐ Non-hydrophilic Silicon catheter ☐ Non-hydrophilic Red rubber (latex) catheter ☐ A combination of different catheter types (please specify) ☐ Other (please specify)

Item 8: What brand of catheter are you currently using?

____

Item 9: What catheter size do you use most of the time (size in French/ Charrier)?

☐ 10 ☐ 12 ☐ 14 ☐ 16

Item 10: What is the shape of the catheter tip?

☐ Catheter with straight tip
☐ Catheter with bent /curved tip (such as a Tiemann catheter)
☐ Catheter with a unidirectional ball tip (such as an IQ or Flex catheter)
☐ Other (please specify)

Item 11: Do you clean your genital area with an antiseptic towelette or wipe before catheterizing?

☐ No ☐ Yes

Item 12: Do you put any lubricant on the catheter before catheterizing?

☐ No ☐ Yes

Section 2. Catheter Reuse

Item 13: Do you reuse your catheter?

☐ No ☐ Yes

Item 14: What are your reasons for reusing?

____

____

____

Item 15: How long have you been reusing your catheters (number of years)?

____

Item 16: What is the shortest amount of time you have ever reused the same catheter (please specify the number of days, weeks or months)?

____

Item 17: What is the longest amount of time you have ever reused the same catheter (please specify the number of days, weeks or months)?

____

Item 18: What is the minimum number of times per day that you reuse the same catheter?

____

Item 19: What is the maximum number of times per day that you reuse the same catheter?

____

Item 20: What methods (if any) do you use to clean/ prepare your catheter for reuse?

____

____

____

Item 21: When, where, and/or how did you learn this cleaning/ preparation method?

____

____

____

Item 22: Have you had any urethral injury(ies) as a result of catheterization after your SCI?

☐ No ☐ Yes

Item 23: During which period(s) did you experience urethral injury(ies) due to catheterization (tick all that apply)?

☐ First year after SCI ☐ 2-5 years after SCI ☐ 6-10 years after SCI ☐ More than 10 years after SCI ☐ Not applicable

Item 24: Have you had any urethral injury(ies) as a result of catheterization by medical personnel?

☐ No ☐ Yes

Item 25: During which period(s) did you experience urethral injury(ies) due to catheterization by medical personnel (tick all that apply)?

☐ First year after SCI ☐ 2-5 years after SCI ☐ 6-10 years after SCI ☐ More than 10 years after SCI ☐ Not applicable

Item 26: Do you ever experience pain in the urethra during catheterization?

☐ No ☐ Yes

Item 27: How often do you experience pain in the urethra during catheterization?

☐ No ☐ Yes

Item 28: Do you sometimes experience difficulties inserting the catheter into the urethra?

☐ No ☐ Yes

Item 29: How often do you experience difficulties inserting the catheter?

☐ Every catheterization ☐ One or more times per week ☐ One or more times per month ☐ One or more times per year ☐ Less than once per year ☐ Not applicable

Item 30: Do you ever notice blood on the catheter after withdrawal from the urethra?

☐ No ☐ Yes

Item 31: How often do you notice blood on the catheter after withdrawal from the urethra?

☐ Every catheterization ☐ One or more times per week ☐ One or more times per month ☐ One or more times per year ☐ Less than once per year ☐ Not applicable

Section 3. Urinary Tract Infections

Item 32: Have you ever had a urinary tract infection (UTI)?

☐ No ☐ Yes

Item 33: How frequently do you get UTIs?

☐ Once per week ☐ Once per month ☐ One or more times per year ☐ Less than once per year ☐ Not applicable

Item 34: What was the approximate date of the last UTI you had?

Item 35: Please describe any additional experiences with regards to having and/or resolving UTIs?

Item 36: Do you use any prophylactic measures (e.g. antibiotics) to avoid getting UTIs? If yes, please specify what you use and how often you use it.

☐ No ☐ Yes

Section 4. Inflammatory Conditions (Males Only)

Item 37: (Males only) Have you ever experienced any inflammation in the testicles?

☐ No ☐ Yes

Item 38: (Males only) During which period(s) did you experience inflammation in the testicles (tick all that apply)?

☐ First year after SCI ☐ 2-5 years after SCI ☐ 6-10 years after SCI ☐ More than 10 years after SCI ☐ Not applicable

Item 39: (Males only) Have you ever experienced any inflammation in the epididymis?

☐ No ☐ Yes

Item 40: (Males only) During which period(s) did you experience inflammation in the epididymis (tick all that apply)?

☐ First year after SCI ☐ 2-5 years after SCI ☐ 6-10 years after SCI ☐ More than 10 years after SCI ☐ Not applicable

Item 41: (Males only) Have you ever experienced any inflammation in the prostate?

☐ No ☐ Yes

Item 42: (Males only) During which period(s) did you experience inflammation in the prostate (tick all that apply)?

☐ First year after SCI ☐ 2-5 years after SCI ☐ 6-10 years after SCI ☐ More than 10 years after SCI ☐ Not applicable

Section 5. Inflammatory Conditions (Females Only)

Item 43: (Females only) Have you ever experienced any pelvic inflammatory conditions?

☐ No ☐ Yes

Item 44: (Females only) During which period(s) did you experience pelvic inflammation (tick all that apply)?

☐ First year after SCI ☐ 2-5 years after SCI ☐ 6-10 years after SCI ☐ More than 10 years after SCI ☐ Not applicable

Supplemental Appendix C. Procedures and assessments

Urine, swab culture and catheter specimen collection

Prior to the start of the trial period, participants visited the laboratory and were briefed on urine, culture swab and catheter specimen collection procedures. Participants were then provided with a package containing all study-related materials.

For culture swab specimen collections, participants were given 9 sterile transport swab tubes (3 per day) (Venturi Transystem™, Copan Diagnostics Inc., Murietta, California, USA), 18 sterile wipes [6 per day to be applied to the genital area before and after each catheterization (optional)] (No-Sting Skin-Prep™, 75% hexamethyldisiloxane, Smith & Nephew Medical Ltd., Hull, England), 9 packets of lubricating jelly [3 per day, to be applied to the length of the catheter to prior to each catheterization (optional)] (3.5g bacteriostatic, non-conductive, water soluble, HealthCare Plus, Toronto, Ontario, Canada), and 9 sterile towels (WypAll™, Kimberley-Clark Worldwide Inc., Mississauga, Ontario, Canada), each inside a separate sealed sterilization pouch (191mm x 330mm, VWR International LLC, Radnor, Pennsylvania, USA) (3 per day, to be used as a sterile surface during the collection of catheter swab culture samples).

For urine sample specimen collections, participants were given 5 sterile urine specimen containers [1 at baseline (i.e., day -2), 3 for each day of the reuse trial and 1 at follow-up (i.e., day 7-15)] (120mL Samco™ Clicktainer™, Thermo Fisher Scientific Inc., Waltham, Massachusetts, USA).

For catheter specimen collection, participants were given 1 pair of sterile surgical scissors with forceps, and a sealed 12cm polyvinyl chloride tube (VWR International LLC, Radnor, Pennsylvania, USA) filled with 45mL of sterile saline solution (0.9% NaCl, Baxter Corp. Mississauga, Ontario, Canada). These were used at the end of the reuse period (i.e., evening of day 3) to clip and store a 10cm section of the reused catheter, inclusive of the tip and eyelets. A diagram summarizing the collection schedule for all urine and culture swab specimens is provided in Figure 1.

Swab gram smear and culture analyses

Gram smear and primary culture analyses were performed by a medical laboratory company (LifeLabs Medical Laboratory Services, OMERS Corporation, Toronto, Ontario, Canada) for all catheter swab specimens. Specimens were labeled and inoculated to plates which were placed onto an automated petri dish streaker (Isoplater 180, Vista Technology Inc. Edmonton, Alberta, Canada). Plate slides were then loaded onto an autostainer (Leica ST5010 Autostainer XL, Leica Biosystems, Deerpark, Illinois, USA) and incubated. Culture growth was correlated with the gram stains and identification testing was performed based on colonial morphology of clinically significant organisms using an appropriate method [i.e., matrix-assisted laser desorption/ionization, Vitek identification cards (Vitek 2 ID cards, bioMérieux Inc., Marcy-l'Étoile, France), or latex agglutination]. Culture plates were then re-incubated for additional growth (i.e., 48hrs). Microbial organism quantity was reported in colony forming units (CFU)/L. Antibiotic resistance and susceptibility for each organism identified were also assessed using an appropriate method [i.e., automated susceptibility testing platform (Vitek 2, bioMérieux Inc., Marcy-l'Étoile, France), Kirby Bauer disk diffusion susceptibility tests, or minimum inhibitory concentration using ETEST (bioMérieux Inc., Marcy-l'Étoile, France)]. No additional susceptibility analyses were performed for cultures containing only skin or mixed flora.

A secondary culture analysis using sonication was conducted for a cohort of 3 randomly selected participants who underwent a second round of reuse with a new catheter in order to verify the reproducibility of culture results obtained from catheter swabs during the primary analysis. Participants reused their catheters for a period of 3 (n=3) and 30 (n=1) consecutive days. Reused and pristine control catheter samples were processed for CFU counts. Reused catheter samples were deposited in sterile containers, and control catheters were kept in their original sterile packages. Control and reused catheters were aseptically removed from their containers, and the first 1.5cm of the tip was cut with a sterile scalpel blade for bacterial counts. Cut catheter pieces were washed in a sterile phosphate-buffered solution (PBS) to remove non-adherent bacteria. Catheter samples were then aseptically transferred to new tubes with fresh PBS and placed in a sonication water bath (Vevor Ultrasonic cleaner, PS 10A, Vevor Inc., Rancho Cucamonga, California, USA) for 10 minutes to remove adherent biofilm/bacteria. The supernatant was serially diluted and 10ul were spot plated on fresh Luria-Bertani

(LB) agar plates in triplicate. Following a 24hr incubation at 37°C, CFU counts were performed to determine the number of bacteria on the catheter surface.

Urinanalysis

Urine culture and sensitivity analyses were performed at the same laboratory testing facility (LifeLabs Medical Laboratory Services, OMERS Corp., Toronto, Ontario, Canada) using the aforementioned procedures. A standardized urine chemistry profile was also generated using a urine analyzer (Clinitek Novus, Siemens Ltd., Munich, Germany). Urine specimens were dispensed onto multiple test pads and then read by the system optical sensor to determine urine clarity, specific gravity, color, pH and the amount of ketone, glucose, protein, hemoglobin, nitrite (i.e., +/-), and leukocytes present in each specimen.

Scanning electron microscopy imaging

Scanning electron microscopy (SEM) imaging of all catheter samples was performed using either a Hitachi SU-3500 system (Hitachi High Technologies Corporation, Tokyo, Japan) or Helios NanoLab 650 Focused Ion Beam system (FEI, Thermo Fisher Scientific, Waltham, Massachusetts, USA) at a 1kV accelerating voltage. For the primary analysis involving 10 catheter samples, SEM imaging was conducted at the Centre for High-Throughput Phenogenomics at the University of British Columbia. Catheter samples were first cut into 2cm length segments, each sliced in two halves using a razor blade. To preserve the structure of the biofilm on catheter surfaces (i.e., bacterial colonization as well as residual debris and encrustation), chemical fixation was applied using 2.5% glutaraldehyde in a 0.1M piperazinediethanesulfonic acid (PIPES) buffered solution (pH 7.4) followed by post fixation in a 0.5% osmium tetroxide solution (pH 6.8). After fixation, samples were dehydrated with ethanol in preparation for critical point drying. Samples were immersed in a series of ethanol-water mixtures of increasing ethanol concentration (ranging from 50-100%, progressively increasing by 10% ethanol concentration for each subsequent solution). Samples were immersed in each solution for 5 minutes. To prevent deformation following dehydration, samples were immediately dried using a Samdri-795 critical point dryer (Tousimis Research Corp., Rockville, Maryland, USA). In order to optimize conductivity, eliminate charging artifact and enhance visualization of structural details during SEM imaging, samples were mounted onto SEM stubs using a quick curing (5 minute) epoxy (System Three Resins, Lacey, Washington, USA) and sputter coated with a thin conductive film of iridium (approximately 8nm) using a precision modular high vacuum coating system (Leica EM MED020, Leica Microsystems Inc., Concord, Ontario, Canada). The samples were fixed to the SEM system stage for positioning and imaging at progressive magnifications (i.e., 25X to 63,500X).

As variance in electron microscopy imaging techniques and interpretation between laboratory settings is expected,[1] a secondary analysis was conducted for catheter samples from the same cohort of participants to examine the reproducibility SEM results following a period of 3 (n=3) and 30 (n=1) consecutive days of catheter reuse. SEM imaging for these samples was performed using a separate Hitachi SU-3500 system (Hitachi High Technologies Corporation, Tokyo, Japan) located at the University of British Columbia. Samples were fixed using a 2.5% glutaraldehyde solution for 1hr and dehydrated using a gradient ethanol/water solution (i.e., from 50% to 100% ethanol). Samples were mounted onto SEM stubs and sputtered coated with iridium (approximately 10nm). Samples were then fixed to the SEM system stage for positioning and imaging at progressive magnifications (i.e., 500X to 5,000X).

X-ray photoelectron spectroscopy analysis

Room-temperature X-ray photoelectron spectroscopy (XPS) was used to examine the surface composition of catheters following 3 (n=3) and 30 (n=1) consecutive days of reuse. All experiments were performed at the nanoFAB Centre of the University of Alberta using an XPS imaging spectrometer (Kratos Axis Ultra, Kratos Analytical Ltd., Manchester, UK) with monochromatized Al Kα (hυ=1486.71 eV). The spectrometer was calibrated using the binding energy (84.0 eV) of Au 4f7/2 with reference to the Fermi level. Analysis chamber pressure during experiments was greater than 5×10-10 Torr. A hemispherical electron-energy analyzer working at a pass energy of 20 eV was used to collect core-level spectra while survey spectrum within a range of binding energies from 0-1100 eV was collected at an analyzer pass energy of 160 eV. Charge effects

were corrected by using C1s peak at 284.8 eV. A Shirley background was applied to subtract the inelastic background of core-level peaks. To determine peak model parameters (i.e., peak positions, widths and intensities) a non-linear optimization using a Marquardt algorithm was performed (CASA XPS software, Surface Analysis Consultation, Clearwater, Florida, USA). The model peak to describe XPS core-level lines for curve fitting was a product of Gaussian and Lorentzian functions and factors provided by the database. A component analysis was performed to fit the spectra of C1s with peaks related to different chemical bonds (CASA XPS software).

References

1. De Temmerman, P.-J.; Verleysen, E.; Lammertyn, J.; Mast, J. Size measurement uncertainties of near-monodisperse, near-spherical nanoparticles using transmission electron microscopy and particle-tracking analysis. *Journal of Nanoparticle Research* **2014**, *16*, 2628, doi:10.1007/s11051-014-2628-3.
